# Synthesis of Regiospecifically Fluorinated Conjugated Dienamides

**DOI:** 10.3390/molecules19044418

**Published:** 2014-04-10

**Authors:** Mohammad Chowdhury, Samir K. Mandal, Shaibal Banerjee, Barbara Zajc

**Affiliations:** Department of Chemistry, The City College and The City University of New York, 160 Convent Avenue, NY 10031, USA

**Keywords:** fluoro dienamides, Julia-Kocienski olefination, Weinreb amide, fluoro dienes

## Abstract

Modular synthesis of regiospecifically fluorinated 2,4-diene Weinreb amides, with defined stereochemistry at both double bonds, was achieved via two sequential Julia-Kocienski olefinations. In the first step, a *Z*-α-fluorovinyl Weinreb amide unit with a benzothiazolylsulfanyl substituent at the allylic position was assembled. This was achieved via condensation of two primary building blocks, namely 2-(benzo[*d*]thiazol-2-ylsulfonyl)-2-fluoro-*N*-methoxy-*N*-methylacetamide (a Julia-Kocienski olefination reagent) and 2-(benzo[*d*]thiazol-2-ylthio)acetaldehyde (a bifunctional building block). This condensation was highly *Z*-selective and proceeded in a good 76% yield. Oxidation of benzothiazolylsulfanyl moiety furnished a *second-generation* Julia-Kocienski olefination reagent, which was used for the introduction of the second olefinic linkage via DBU-mediated condensations with aldehydes, to give (2*Z*,4*E*/*Z*)-dienamides in 50%–74% yield. Although olefinations were 4*Z*-selective, (2*Z*,4*E*/*Z*)-2-fluoro-2,4-dienamides could be readily isomerized to the corresponding 5-substituted (2*Z*,4*E*)-2-fluoro-*N*-methoxy-*N*-methylpenta-2,4-dienamides in the presence of catalytic iodine.

## 1. Introduction

**Figure 1:**
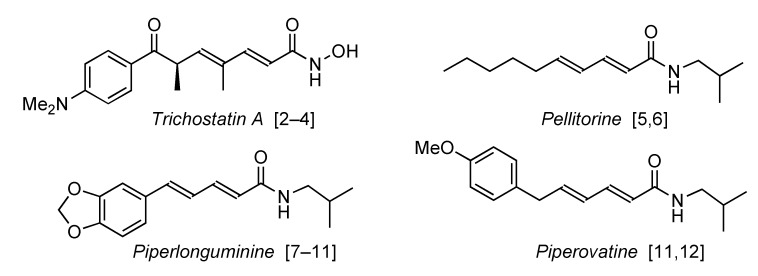
Examples of naturally occurring, biologically active 2,4-dienamides.

The conjugated diene and polyene amide structural units are found in many naturally occurring compounds that possess biological activity [1]. These compounds have a variety of uses, ranging from medicinal purposes, to insecticides, as well as culinary flavoring agents [1]. Some examples of dienamides are shown in Figure 1. Trichostatin A is an antifungal antibiotic [2], and as an inhibitor of mammalian histone deacetylase [3], is a potential anticancer agent [4]. Pellitorine has insecticidal [5] and cytotoxic [6] activities. Piperlonguminine has broad-ranging therapeutic activities [7] such as antibacterial, antifungal, antitumor [8], anticoagulant [9], antimelanogenesis [10], and anti-inflammatory [11], to name a few. Piperovatine exhibits local anesthetic [12] and antinflammatory activities [11]. Due to their interesting biological activities, several analogs, such as a fluorinated Trichostatin A analog [13], have been synthesized as well.

Fluorine is an attractive substituent in pharmaceuticals, in agrochemicals, and in materials chemistry [14,15,16] due to its effect on physical, chemical, and biological properties of compounds [17,18,19]. Julia-Kocienski olefinations [20,21,22,23] have been explored for the synthesis of various functionalized fluoroolefins [24,25,26] by us [27,28,29,30,31,32,33,34,35] and by others [36,37,38,39,40,41,42,43,44,45,46]. In the course of our recent work, we became interested in the use of bifunctional Julia-Kocienski reagents, or their precursors, for novel modular assembly of vinyl [33,47] and fluorovinyl [33] compounds. Herein, we report the synthesis of regiospecifically fluorinated dienamides, via sequential olefination of a bifunctional Julia-Kocienski building block. As the amide functionality, we chose the Weinreb amide, both to test the feasibility of the methodology and due to its versatility via its unique reactivity properties [48,49,50,51].

## 2. Results and Discussion

**Scheme 1:**
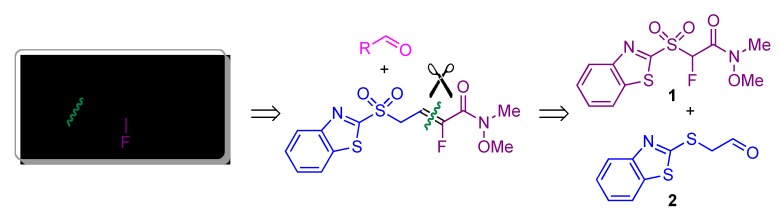
Retrosynthetic analysis for the preparation of conjugated dieneamides.

Key to our approach was the assembly of a α-fluorovinyl Weinreb amide moiety with functionality at the beta position that could be used in a sequential condensation. We have previously reported the synthesis and studied the reactivity of a Julia-Kocienski reagent for the preparation of α-fluorovinyl Weinreb amides (**1**, Scheme 1) [31]. Condensation of 2-(benzo[*d*]thiazol-2-ylsulfonyl)-2-fluoro-*N*-methoxy-*N*-methylacetamide (**1**) with a 2-(heteroarylthio)ethanal (**2**, heteroaryl = benzothiazolyl, Scheme 1) and subsequent oxidation would furnish a second-generation Julia-Kocienski reagent for dienamide synthesis. A retrosynthetic approach to conjugated dieneamides is outlined in Scheme 1.

The requisite Julia-Kocienski reagent, 2-(benzo[*d*]thiazol-2-ylsulfonyl)-2-fluoro-*N*-methoxy-*N*-methylacetamide (**1**), was synthesized as reported [31]. Synthesis of the other key reactive partner, 2-(benzo[*d*]thiazol-2-ylthio)acetaldehyde (**2**), was initially attempted via the dioxolane derivative of **2**. Although the dioxolane derivative of **2** could be readily prepared from 2-(bromomethyl)-1,3-dioxolane and the sodium salt of 2-mercapto-1,3-benzothiazole, attempts at deprotection of 2-[(1,3-dioxolan-2-yl)methylthio]benzo[*d*]thiazole under various conditions proved unsuccessful. Therefore, synthesis via the dimethyl acetal was considered (Scheme above Table 1).

**Table 1:** Synthesis of 2-(benzo[*d*]thiazol-2-ylthio)acetaldehyde **2**. 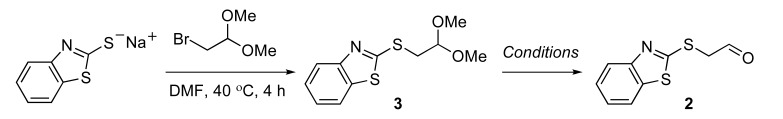

Entry	Reagent	Solvent	T (°C)	Time	Yield (%) *^a^*
1	I_2_	acetone	rt	overnight	-- *^b^*
2	CBr_4_	CH_3_CN–H_2_O 1:3	80	3 days	-- *^b^*
3	PTSA	THF–H_2_O 1:1	rt	3 days	-- *^b^*
4	HCl (4 M)	acetone	40	24 h	20 *^c^*
5	HCl (4 M)	acetone	40	4 h	56
6	HCl (12 M)	acetone	50	30 min	81
7	HCl (12 M)	acetone–H_2_O 10:1	50	40 min	84

*^a^* Aldehyde **2** was unstable under chromatographic conditions, either on silica gel or on alumina. Therefore, the yield reported for **2** is without purification, unless stated otherwise; *^b^* ^1^H-NMR and TLC showed only dimethyl acetal **3**, and no product formation was observed; *^c^* Isolated yield after column chromatography.

Various conditions were tested to unmask the aldehyde functionality (Table 1). Upon reaction of dimethyl acetal **3** with I_2_ (entry 1), CBr_4_ (entry 2), or PTSA (entry 3), no hydrolysis was observed. Reaction of **3** with 4 M HCl at 40 °C resulted in complete consumption of **3**, but aldehyde **2** was isolated in a low 20% yield after column chromatography (entry 4). Subsequently, we found that compound **2** is unstable under chromatography conditions, on silica gel and alumina [52]. The yield of crude **2** after hydrolysis with 4 M HCl, but without chromatography, was 56%. Hydrolysis with 12 M HCl at 50 °C was complete within 30 min, yielding crude **2** in 81% yield (entry 6). However, due to the solubility of **2** in water, we obtained inconsistent results in repeat experiments. After extensive experimentation we found that crude **2** could be isolated in consistent yields when aqueous workup was avoided. Briefly, acetal **3** was reacted with 12 M HCl in acetone–H_2_O (10:1) at 50 °C for 40 min (entry 7), solid NaHCO_3 _was added portion-wise at 5 °C to neutralize the acid, and excess water was removed by addition of anhydrous Na_2_SO_4_. The solution was then passed through a bed of anhydrous Na_2_SO_4_ and the solvent was evaporated to afford **2** in >80% yield. When acetone alone was used as solvent, complete hydrolysis of **3** occurred, but the crude product showed the presence of an unidentified byproduct that could possibly result from the condensation of acetone and **2**. The use of water as a co-solvent therefore seems to be crucial in order to minimize the formation of the byproduct.

With both desired building blocks in hand, *i.e.*, the Julia-Kocienski reagent **1** and aldehyde **2**, we tested reaction conditions for the olefination reaction (Table 2). All condensation reactions were performed at −78 °C in the presence of LHMDS, and gave (*Z*)-4-(benzo[*d*]thiazol-2-ylthio)-2-fluoro-*N*-methoxy-*N*-methylbut-2-enamide (**4**) as the only stereoisomer. Comparably, exclusive *Z*-selectivity has also been observed in NaH-mediated condensations of **1** with aldehydes [31]. In the reactions herein, the molar ratio of sulfone **1**, aldehyde **2**, and LHMDS was critical for obtaining a good yield of **4** (Table 2). When aldehyde **2** was used as a limiting reactant (entry 1), or in an equimolar amount (entry 2), enamide **4** was obtained in low yield. On the other hand, with excess aldehyde **2** and LHMDS, a substantial yield improvement was observed. Thus, product **4** was isolated in 76% yield when 2 molar equiv of **2** and 3 molar equiv of LHMDS were used (entry 4). Since the desired product was obtained with exclusive *Z*-selectivity and in a good yield, we did not attempt to use other bases, such as KHMDS or NaHMDS.

**Table 2:** Synthesis of (*Z*)-4-(benzo[*d*]thiazol-2-ylthio)-2-fluoro-*N*-methoxy-*N*-methylbut-2-enamide (**4**). 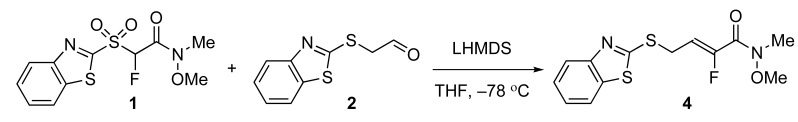

Entry	Molar Ratio of 1:2:LHMDS	Time	Yield (%) *^a^*
1	1.5:1:1.5	3 h	32
2	1:1:2	2.5 h	20
3	1:3:5	4 h	60
4	1:2:3	3.5 h	76

*^a^* Yield is of isolated and purified product **4**. Reactions were monitored for completion by ^19^F-NMR. LHMDS was added portion-wise (please see Experimental Section).

In order to obtain the *second generation* Julia-Kocienski reagent **5**, sulfide **4** was oxidized using H_5_IO_6_ and catalytic CrO_3_. Sulfone **5**, obtained in 63% yield, was then used for the screening of reaction conditions for the olefination with 2-naphthaldehyde (Table 3).

**Table 3:** Conditions tested for olefination reactions using the *second generation* Julia-Kocienski reagent **5** and 2-naphthaldehyde. 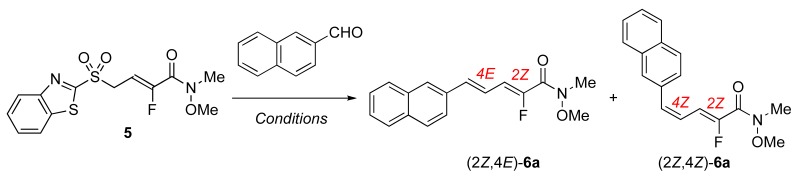

Entry	Base	Solvent	T	Time	% 4*E*/4*Z* Ratio *^a^*	Yield (%) *^b^*
1	LHMDS	THF	−78 to 0 °C	overnight	--	-- *^c^*
2	LHMDS	THF	0 °C to rt	12 h	--	-- *^c^*
3	DBU	THF	rt	2 h	--	-- *^c^*
4	DBU	THF	−78 to 0 °C	overnight	57/43	35
5	Cs_2_CO_3_	THF	0 °C	overnight	--	-- *^c^*
6	DBU	THF	0 °C	overnight	43/57	55
7	Cs_2_CO_3_	CH_2_Cl_2_	0 °C	overnight	--	-- *^c^*
8	DBU	CH_2_Cl_2_	0 °C	overnight	35/65	66

*^a^* The relative ratio of isomers in the crude reaction mixtures was determined by ^19^F-NMR prior to isolation. No change in the relative ratio was observed after purification; *^b^* Yield is of isolated and purified product 6a; *^c^* No product was detected either by ^19^F-NMR or by TLC.

Both selectivity and product yield depended upon the reaction conditions. No product formation occurred when LHMDS was used as base (entries 1 and 2), or with DBU as base in THF at room temperature (entry 3). Similarly, Cs_2_CO_3_ in either THF or CH_2_Cl_2_ at 0 °C did not show product formation (entries 5 and 7). Product **6a** was obtained in a low 35% yield and with a moderate 4*E* selectivity in an overnight reaction with DBU in THF, at −78 to 0 °C (*E*/*Z* 57/43, entry 4). When the condensation reaction was allowed to run overnight at 0 °C (entry 6), product **6a** was isolated in a better 55% yield, but with a reversed selectivity as compared to entry 4 (*E*/*Z* 43/57). Yield and selectivity increased when the condensation reaction was performed overnight using DBU as base in CH_2_Cl_2_, at 0 °C (66%, entry 8).

Using these conditions, the generality of condensation reactions of Julia-Kocienski reagent **5** with other aldehydes was tested. Table 4 shows yields, the 4*E*/4*Z* ratios, and ^19^F-NMR data of the products.

**Table 4:** Reactions of reagent **5** with aldehydes: yields, *E*/*Z* ratios, and ^19^F-NMR data. 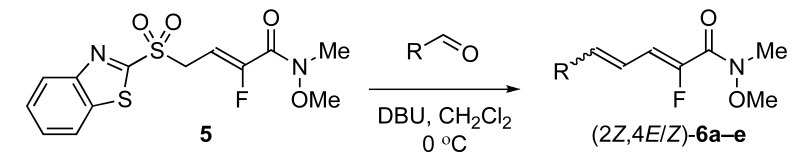

Entry	RCHO	Product (6a–e); % 4*E*/4*Z* Ratio *^a^*; Yield (%) *^b^*	^19^F-NMR Data: *^c^* *δ* (ppm); Mult, *J* (Hz)
1	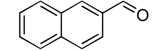	**6a**: 35/65; 66	(4*E*)-**6a**: −123.4; d, 30.5 (4*Z*)-**6a**: −121.2; d, 30.5
2	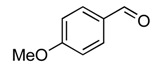	**6b**: 23/77; 50	(4*E*)-**6b**: −125.2; d, 33.6 (4*Z*)-**6b**: −122.6; d, 33.6
3	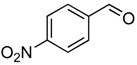	**6c**: 40/60; 74	(4*E*)-**6c**: −119.6; d, 30.5 (4*Z*)-**6c**: −118.4; d, 30.5
4	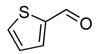	**6d**: 10/90; 63	(4*E*)-**6d**: −123.7; d, 30.5 (4*Z*)-**6d**: −120.7; d, 33.6
5	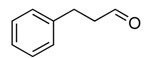	**6e**: 15/85; 51	(4*E*)-**6e**: −125.7; d, 30.5 (4*Z*)-**6e**: −124.2; d, 33.6

*^a^* The relative ratio of isomers in the crude reaction mixtures was determined by ^19^F-NMR prior to isolation; *^b^* Yield is of isolated and purified product **6**; *^c^* ^19^F-NMR spectra were recorded at 282 MHz, in CDCl_3_ with CFCl_3_ as an internal reference.

Moderate to high 4*Z* selectivity was obtained with electron-rich aryl and heteroaryl aldehydes, with yields ranging from 50%–66% (entries 1, 2 and 4). The electron-deficient *p*-nitrobenzaldehyde gave product **6c** in a good 74% yield, but with poor 4*Z* selectivity (entry 3). Reaction of **5** with 3-phenylpropanal gave product **6e** in a moderate 51% yield and with high 4*Z* selectivity (entry 5). In the ^19^F-NMR spectra of all products, the doublet from the (4*E*)-isomer appears more upfield as compared to the doublet from the (4*Z*)-isomer (Table 4, entries 1–5).

Next, we considered isomerization of the (2*Z,*4*Z*)-isomer to the (2*Z,*4*E*)-isomer. Several techniques were evaluated to effect this isomerization. Overnight exposure of the 4*E*/4*Z* isomer mixture to light (20 watt bulb) did not cause any isomerization. Treatment of the isomer mixtures with silica powder in CHCl_3 _ at room temperature or at 0 °C showed the desired isomerization, but the isomerization did not proceed to completion. A convenient method for the isomerization using catalytic I_2_ in CHCl_3_ at room temperature has been reported [53]. Using this method, complete isomerization of (2*Z*,4*E*/*Z*)-**6a**–**d** to (2*Z*,4*E*)-**6a**–**d** was achieved (Table 5).

**Table 5:** Isomerization of (2*Z*,4*E*/*Z*)-**6a**–**d** to (2*Z*,4*E*)-**6a**–**d**. 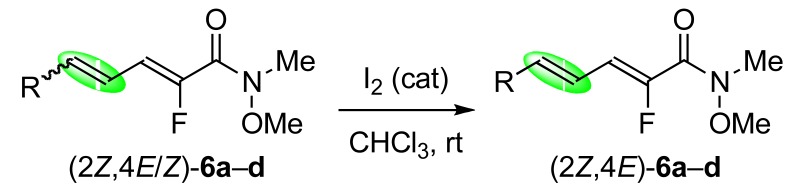

Entry	Isomer Mixture	Time	Product *^a^*	Yield (%) *^b^*
1	(2*Z*,4*E*/*Z*)-**6a**	3 h	(2*Z*,4*E*)-**6a**	75
2	(2*Z*,4*E*/*Z*)-**6b**	3 h	(2*Z*,4*E*)-**6b**	86
3	(2*Z*,4*E*/*Z*)-**6c**	1.5 h	(2*Z*,4*E*)-**6c**	89
4	(2*Z*,4*E*/*Z*)-**6d**	overnight	(2*Z*,4*E*)-**6d**	92

*^a^* Olefin geometry was determined by ^1^H-NMR; *^b^* Yield is of the isolated and purified isomer.

## 3. Experimental

### 3.1. General Information

THF was distilled over LiAlH_4_ and then over sodium. CH_2_Cl_2_, EtOAc, and hexanes were distilled over CaCl_2_. For reactions performed under a nitrogen atmosphere, glassware was dried with a heat gun under vacuum. LHMDS (1.0 M in THF) was obtained from commercial sources. Julia Kocienski reagent 2-(benzo[*d*]thiazol-2-ylsulfonyl)-2-fluoro-*N*-methoxy-*N*-methylacetamide (**1**) was prepared from the known 2-(benzo[*d*]thiazol-2-ylsulfonyl)-*N*-methoxy-*N*-methylacetamide [54], via metalation-fluorination using our previously reported procedure [31]. All other reagents were obtained from commercial sources and used without further purification. Thin layer chromatography was performed on Analtech silica gel plates (250 μm). Column chromatographic purifications were performed on 200–300 mesh silica gel. ^1^H-NMR spectra were recorded at 500 MHz in CDCl_3_ and are referenced to residual solvent. ^13^C-NMR spectra were recorded at 125 MHz and are referenced to the carbon resonance of the deuterated solvent. ^19^F-NMR spectra were recorded at 282 MHz with CFCl_3_ as an internal standard. Chemical shifts (*δ*) are reported in parts per million and coupling constants (*J*) are in hertz (Hz).

### 3.2. Synthesis of “Second-Generation” Julia-Kocienski Reagent **5**

*2-(2,2-Dimethoxyethylthio)benzo[d]thiazole* **3***.* To a solution of the sodium salt of 2-mercapto-1,3-benzothiazole (1.67 g, 8.83 mmol, 1.49 molar equiv.) in DMF (20 mL) was added 2-bromo-1,1-dimethoxyethane (1.00 g, 5.91 mmol), and the mixture was allowed to stir at 40 °C for 4 h. Upon completion of the reaction, as observed by TLC, the reaction mixture was diluted with EtOAc and washed with water. The aqueous layer was extracted with EtOAc (3 × 30 mL). The combined organic layer was washed with saturated NaHCO_3_ (30 mL), brine, dried over anhydrous Na_2_SO_4_, and evaporated. The crude product was purified by column chromatography using 20% EtOAc in hexanes to obtain compound 3 (0.769 g, 51%) as a colorless viscous liquid. R_f_ (SiO_2_, 20% EtOAc in hexanes): 0.48. ^1^H-NMR (CDCl_3_): *δ* 7.85 (d, 1H, Ar-H, *J* = 7.8 Hz), 7.75 (d, 1H, Ar-H, *J* = 8.3 Hz), 7.41 (t, 1H, Ar-H, *J* = 7.8 Hz), 7.29 (t, 1H, Ar-H, *J* = 7.8 Hz), 4.72 (t, 1H, *J* = 5.3 Hz), 3.58 (d, 2H, *J* = 5.3 Hz), 3.44 (s, 6H, OCH_3_). ^13^C-NMR (CDCl_3_): *δ* 166.4, 153.2, 135.5, 126.1, 124.4, 121.6, 121.1, 103.0, 54.3, 35.5. HRMS (ESI) calcd for C_11_H_14_NO_2_S_2_ [M+H]^+^ 256.0460, found 256.0462.

*2-(Benzo[d]thiazol-2-ylthio)acetaldehyde* **2**. To a stirred solution of 2-(2,2-dimethoxyethylthio)benzo[*d*]thiazole (**3**, 1.60 g, 6.26 mmol) in acetone (48 mL), was slowly added a mixture of HCl (12 M, 10.6 mL) and water (5.2 mL) at rt. The mixture was stirred for 40 min at 50 °C. Upon completion of the reaction, as observed by TLC, the mixture was cooled to 5 °C and the reaction was quenched by portion-wise addition of solid NaHCO_3_ up to the neutralization point, and then passed through a bed of anhydrous Na_2_SO_4_. The anhydrous Na_2_SO_4_ bed was washed with a minimum amount of acetone, the combined eluent was dried over anhydrous Na_2_SO_4_, and concentrated under reduced pressure. Crude product **2** (1.10 g, 84%) was used in the next step without purification. R_f_ (SiO_2_, 20% EtOAc in hexanes): 0.29. ^1^H-NMR (CDCl_3_): *δ* 9.73 (br, 1H), 7.85 (d, 1H, Ar-H, *J* = 7.9 Hz), 7.76 (d, 1H, Ar-H, *J* = 8.2 Hz), 7.42 (t, 1H, Ar-H, *J * = 7.6 Hz), 7.32 (t, 1H, Ar-H, *J * = 7.6 Hz), 4.09 (d, 2H, *J * = 1.8 Hz).

*(Z)-4-(Benzo[d]thiazol-2-ylthio)-2-fluoro-N-methoxy-N-methylbut-2-enamide* **4**. To a stirred solution of 2-(benzo[*d*]thiazol-2-ylsulfonyl)-2-fluoro-*N*-methoxy-*N*-methylacetamide (**1**, 0.700 g, 2.20 mmol) and 2-(benzo[*d*]thiazol-2-ylthio)acetaldehyde (**2**, 0.930 g, 4.44 mmol, 2.0 molar equiv.) in dry THF (48.0 mL) at −78 °C (dry ice/iPrOH), was added LHMDS (4.39 mL, 1 *M*, 4.39 mmol, 2.0 molar equiv.) dropwise under a nitrogen atmosphere. The mixture was allowed to stir at −78 °C (dry ice/iPrOH) for 2 h and checked for the disappearance of **1** by ^19^F-NMR (a small sample was removed by syringe and checked by NMR). Since ^19^F-NMR showed the presence of **1**, more LHMDS (2.19 mL, 1 M, 2.19 mmol, 1 molar equiv.) was added and the mixture was allowed to stir at −78 °C for an additional 1 h at which time complete consumption of sulfone **1** was observed by ^19^F-NMR. The reaction was quenched by the addition of saturated aq. NH_4_Cl, the solvent was partially removed under reduced pressure, and the mixture was extracted with EtOAc (3×). The combined organic layer was washed with 5% aq. NaOH, followed by water and brine, and then dried over Na_2_SO_4_. The organic layer was concentrated under reduced pressure and the crude product was purified by column chromatography using 10%, 15%, and 20% EtOAc in hexanes, to afford compound **4** as a yellow wax (0.523 g, 76%). R_f_ (SiO_2_, 30% EtOAc in hexanes): 0.54. ^1^H-NMR (CDCl_3_): *δ* 7.87 (d, 1H, Ar-H, *J * = 8.3 Hz), 7.75 (d, 1H, Ar-H, *J * = 7.8 Hz), 7.43–7.40 (m, 1H, Ar-H), 7.32–7.28 (m, 1H, Ar-H), 6.22 (dt, 1H, *J* = 32.7; 8.0 Hz), 4.15 (dd, 2H, *J * = 8.0; 1.5 Hz), 3.69 (s, 3H, OCH_3_), 3.22 (s, 3H, CH_3_). ^13^C-NMR (CDCl_3_): *δ* 165.2, 161.8 (d, *J*_CF_ = 27.9 Hz), 153.3, 152.4 (d, *J*_CF_ = 271.9 Hz), 135.7, 126.3, 124.6, 121.9, 121.2, 113.0 (d, *J*_CF_ = 10.5 Hz), 62.1, 33.9, 26.9 (d, *J*_CF _ = 5.5 Hz). ^19^F-NMR (CDCl_3_): *δ* −119.9 (d, ^3^*J*_HF_ = 30.5 Hz). HRMS (ESI) calcd for C_13_H_14_FN_2_O_2_S_2_ [M+H]^+^ 313.0475, found 313.0480.

*(Z)-4-(Benzo[d]thiazol-2-ylsulfonyl)-2-fluoro-N-methoxy-N-methylbut-2-enamide* **5**. H_5_IO_6_ (0.477 g, 2.09 mmol, 3.0 molar equiv) was dissolved in CH_3_CN (80.0 mL) by vigorous stirring at rt for 20 min. CrO_3_ (25.0 mg, 0.25 mmol) was added and the reaction mixture was stirred for an additional 5 min to give an orange-colored solution. A solution of (*Z*)-4-(benzo[*d*]thiazol-2-ylthio)-2-fluoro-*N*-methoxy-*N*-methybut-2-enamide (**4**, 0.218 g, 0.698 mmol) in CH_3_CN (10.0 mL) was added dropwise to this mixture, resulting in an exothermic reaction and the formation of a yellowish precipitate. After complete addition, the mixture was stirred for 3 h, at which time TLC showed complete consumption of amide **4**. The mixture was filtered through a Celite pad, the pad was washed with CH_3_CN, and the filtrate was concentrated under reduced pressure. Water was added to the residue and the mixture was extracted with EtOAc (3 × 30 mL). The combined organic layer was washed with saturated aq. NaHCO_3_ (5 × 30 mL) and brine (30 mL), and dried over anhydrous Na_2_SO_4_. The organic layer was concentrated under reduced pressure and the crude product was purified by column chromatography using 15%, 20%, and 25% EtOAc in hexanes to afford compound 5 as a white solid (0.152 g, 63%). R_f_ (SiO_2_, 40% EtOAc in hexanes): 0.43. ^1^H-NMR (CDCl_3_): *δ* 8.24 (d, 1H, Ar-H, *J * = 7.8 Hz), 8.02 (d, 1H, Ar-H, *J * = 7.8 Hz), 7.67–7.59 (m, 2H, Ar-H), 5.96 (dt, 1H, *J * = 31.2; 8.3 Hz), 4.43 (dd, 2H, *J * = 8.3; 1.5 Hz), 3.53 (s, 3H, OCH_3_), 3.17 (s, 3H, CH_3_). ^13^C-NMR (CDCl_3_): *δ* 164.8, 160.8 (d, *J*_CF_ = 28.4 Hz), 155.6 (d, *J*_CF_ = 279.7 Hz), 152.8, 137.2, 128.4, 127.9, 125.8, 122.6, 102.7 (d, *J*_CF_ = 10.1 Hz), 62.1, 51.1 (d, *J*_CF_ = 4.6 Hz), 33.6. ^19^F-NMR (CDCl_3_): *δ* −113.6 (d, ^3^*J*_HF _*= * 30.5 Hz). HRMS (ESI) calcd for C_13_H_14_FN_2_O_4_S_2_ [M+H]^+^ 345.0374, found 345.0376.

### 3.3. Condensation Reactions of Julia-Kocienski Reagent **5**

*General experimental procedure.* To a stirred solution of aldehyde (0.20 mmol) in dry CH_2_Cl_2_ (10.0 mL) was added DBU (121.7 mg, 0.80 mmol, 4.0 molar equiv.) and the mixture was cooled to 0 °C. A solution of (*Z*)-4-(benzo[*d*]thiazol-2-ylsulfonyl)-2-fluoro-*N*-methoxy-*N*-methylbut-2-enamide (**5**, 103.3 mg, 0.300 mmol, 1.5 equiv.) in dry CH_2_Cl_2_ (10.0 mL) was then added slowly, dropwise (over about 2 h). The reaction mixture was allowed to stir overnight at 0 °C. After completion of the reaction, the solvent was evaporated under a stream of nitrogen gas, and the ^1^H and ^19^F-NMR spectra of the crude product mixture were recorded for determination of the *E*/*Z *ratio. The combined *E*/*Z *product mixture was purified by column chromatography. For eluting solvents see the specific compound headings. The mixture of (4*E*)*-* and (4*Z*)-isomers was analyzed and characterized based on the ^1^H-NMR; the assignment and integration of specific olefinic proton(s) allowed for other signals to be assigned based on the integration, along with a comparison to the pure (2*Z*,4*E*)-isomer (obtained after isomerization, *vide infra*). Assignment of the ^19^F-NMR signals to the (4*E*)- and (4*Z*)-isomers was based on the integration.

*(2Z,4E/Z)-2-Fluoro-N-methoxy-N-methyl-5-(naphthalen-2-yl)penta-2,4-dienamide* **6a**. Isomer ratio of (4*E*)-**6a**:(4*Z*)-**6a** = 35:65. Column chromatography using 8%, 10%, and 15% EtOAc in hexanes gave a mixture of (2*Z*,4*E/Z*)-**6a** as a white solid (38.0 mg, 66%). R_f_ (SiO_2_, 30% EtOAc in hexanes): 0.49 for (4*E*)-**6a** and 0.58 for (4*Z*)-**6a**. ^1^H-NMR (CDCl_3_): *δ* 7.85–7.80 (m, Ar-H, 4H, (4*E*)*-*isomer and 4H, (4*Z*)*-*isomer), 7.68 (dd, 1H, Ar-H, *J * = 8.3; 1.5 Hz, (4*E*)-isomer), 7.50–7.45 (m, Ar-H, 2H, (4*E*)*-*isomer and 3H, (4*Z*)*-*isomer), 7.21 (dd, 1H, *J * = 15.6; 11.2 Hz, (4*E*)*-*isomer), 7.06 (ddd, 1H, *J * = 32.7; 12.0; 1.0 Hz, (4*Z*)*-*isomer), 6.97 (d, 1H, *J* = 15.6 Hz, (4*E*)*-*isomer), 6.91 (d, 1H, *J * = 11.2 Hz, (4*Z*)*-*isomer), 6.72 (dd, 1H, *J* = 32.5; 11.5 Hz, (4*E*)*-*isomer), 6.66 (t, 1H, *J * = 11.7 Hz, (4*Z*)-isomer), 3.80 (s, 3H, OCH_3_, (4*E*)*-*isomer), 3.76 (s, 3H, OCH_3_, (4*Z*)*-*isomer), 3.29 (s, 3H, CH_3_, (4*E*)*-*isomer), 3.25 (s, 3H, CH_3_, (4*Z *)isomer). ^19^F-NMR (CDCl_3_): *δ* –121.2 (d, ^3^*J*_HF_ = 30.5 Hz, (4*Z*)*-*isomer), −123.4 (d, ^3^*J*_HF_ = 30.5 Hz, (4*E*)*-*isomer). HRMS (ESI) calcd for C_17_H_17_FNO_2_ [M+H]^+^ 286.1238, found 286.1242.

*(2Z,4E/Z)-2-Fluoro-N-methoxy-N-methyl-5-(4-methoxyphenyl)penta-2,4-dienamide* **6b**. Isomer ratio of (4*E*)-**6b**:(4*Z*)-**6b** = 23:77. Column chromatography using 8%, 15%, and 20% EtOAc in hexanes gave a mixture of (2*Z*,4*E/Z*)-**6b** as a pale yellow solid (26.5 mg, 50%). R_f_ (SiO_2_, 30% EtOAc in hexanes): 0.35. ^1^H-NMR (CDCl_3_): *δ* 7.42 (d, 2H, Ar-H, *J* = 8.8 Hz, (4*E*)-isomer), 7.29 (d, 2H, Ar-H, *J* = 8.8 Hz, (4*Z*)*-*isomer), 6.99 (ddd, 1H, *J* = 32.7; 11.7; 0.9 Hz, (4*Z*)-isomer), 6.95 (dd, 1H, *J* = 15.6; 11.2 Hz, (4*E*)-isomer), 6.91–6.87 (m, Ar-H, 2H, (4*Z*)-isomer and 2H, (4*E*)-isomer), 6.75 (d, 1H, *J* = 15.6 Hz, (4*E*)-isomer), 6.69 (br d, 1H, *J* = 11.7 Hz, (4*Z*)-isomer), 6.65 (dd, 1H, *J* = 32.7; 11.4 Hz, (4*E*)-isomer), 6.47 (t, 1H, *J* = 11.7 Hz, (4*Z*)-isomer), 3.83 (s, 3H, OCH_3_, (4*E*)-isomer), 3.82 (s, 3H, OCH_3_, (4*Z*)*-*isomer), 3.77 (s, 3H, OCH_3_, (4*E*)-isomer), 3.76 (s, 3H, OCH_3_, (4*Z*)-isomer), 3.27 (s, 3H, CH_3_, (4*E*)-isomer), 3.25 (s, 3H, CH_3_, (4*Z*)-isomer). ^19^F-NMR (CDCl_3_): *δ* −122.6 (d, ^3^*J*_HF_ = 33.6, (4*Z*)*-*isomer), −125.2 (d, ^3^*J*_HF_ = 33.6, (4*E*)*-*isomer). HRMS (ESI) calcd for C_14_H_17_FNO_3_ [M+H]^+^ 266.1187, found 266.1191.

*(2Z,4E/Z)-2-Fluoro-N-methoxy-N-methyl-5-(4-nitrophenyl)penta-2,4-dienamide* **6c**. Isomer ratio of (4*E*)-**6c**:(4*Z*)-**6c** = 4:6. Column chromatography using 10% and 15% EtOAc in hexanes gave a mixture of (2*Z*,4*E/Z*)-**6c** as a yellow solid (41.3 mg, 74%). R_f_ (SiO_2_, 30% EtOAc in hexanes): 0.47 for (4*E*)*-***6c** and 0.72 for (4*Z*)*-***6c**. ^1^H-NMR (CDCl_3_): *δ* 8.23 (d, 2H, Ar-H, *J* = 8.8 Hz, (4*Z*)*-*isomer), 8.21 (d, 2H, Ar-H, *J* = 8.7 Hz, (4*E*)-isomer), 7.60 (d, 2H, Ar-H, *J* = 8.8 Hz, (4*E*)-isomer), 7.48 (d, 2H, Ar-H, *J* = 8.3 Hz, (4*Z*)-isomer), 7.23 (dd, 1H, *J* = 15.9; 11.5 Hz, (4*E*)*-*isomer), 6.86–6.61( m, 3H, (4*Z*)-isomer and 2H, (4*E*)-isomer), 3.79 (s, 3H, OCH_3_, (4*E*)-isomer), 3.77 (s, 3H, OCH_3_, (4*Z*)-isomer), 3.28 (s, 3H, CH_3_, (4*E*)-isomer), 3.25 (s, 3H, CH_3_, (4*Z*)-isomer). ^19^F-NMR (CDCl_3_): *δ* −118.4 (d, ^3^*J*_ HF_ = 30.5 Hz, (4*Z*)-isomer), −119.6 (d, ^3^*J*_ HF_ = 30.5 Hz, (4*E*)-isomer). HRMS (ESI) calcd for C_13_H_14_FN_2_O_4_ [M+H]^+^ 281.0932, found 281.0935.

*(2Z,4E/Z)-2-Fluoro-N-methoxy-N-methyl-5-(thiophen-2-yl)penta-2,4-dienamide* **6d**. Isomer ratio of (4*E*)-**6d**:(4*Z*)-**6d** = 1:9. Column chromatography using 8% and 10% EtOAc in hexanes gave a mixture of (2*Z*,4*E/Z*)-**6d** as a yellow solid (30.2 mg, 63%). R_f_ (SiO_2_, 30% EtOAc in hexanes): 0.34 for (4*E*)-**6d** and 0.37 for (4*Z*)*-***6d**. ^1^H-NMR (CDCl_3_): *δ* 7.36 (d, 1H, Ar-H, *J* = 4.9 Hz, (4*Z*)-isomer), 7.27 (ddd, 1H, *J* = 31.7; 12.2; 1.0 Hz, (4*Z*)-isomer), 7.27–7.25 (overlapping with CHCl_3_, 1H, Ar-H, (4*E*)-isomer), *7*.12 (d, 1H, Ar-H, *J* = 3.4 Hz, (4*Z*)-isomer), 7.09 (d, 1H, Ar-H, *J* = 3.4 Hz, (4*E*)-isomer), 7.04 (dd, 1H, Ar-H, *J* = 5.1; 3.7 Hz, (4*Z*)-isomer), 7.00 (dd, 1H, Ar-H, *J* = 5.1; 3.7 Hz, (4*E*)-isomer), 6.92 (d, 1H, *J* = 15.6 Hz, (4*E*)-isomer), 6.86 (dd, 1H, *J* = 15.6; 10.7 Hz, (4*E*)-isomer), 6.76 (br d, 1H, *J* = 11.2 Hz, (4*Z*)-isomer), 6.61 (dd, 1H, *J* = 32.2; 10.7 Hz, (4*E*)-isomer), 6.42 (t, 1H, *J* = 12.0 Hz, (4*Z*)-isomer), 3.78 (s, 3H, OCH_3_, (4*Z*)-isomer), 3.77 (s, 3H, OCH_3_, (4*E*)*-*isomer), 3.28 (s, 3H, CH_3_, (4*Z*)-isomer), 3.26 (s, 3H, CH_3_, (4*E*)*-*isomer). ^19^F-NMR (CDCl_3_): *δ* −120.7 (d, ^3^*J*_HF_ = 33.6 Hz, (4*Z*)-isomer), −123.7 (d, ^3^*J*_HF_ = 30.5 Hz, (4*E*)-isomer). HRMS (ESI) calcd for C_11_H_13_FNO_2_S [M+H]^+^ 242.0646, found 242.0647.

*(2Z,4E/Z)-2-Fluoro-N-methoxy-N-methyl-7-phenylhepta-2,4-dienamide 6e*. Isomer ratio of (4*E*)-**6e**:(4*Z*)-**6e** = 15:85. Column chromatography using 6%, 8%, and 12% EtOAc in hexanes gave a mixture of (2*Z*,4*E/Z*)-**6e** as a colorless oil (26.6 mg, 51%). R_f_ (SiO_2_, 30% EtOAc in hexanes): 0.30. We were unable to resolve the ^1^H-NMR signals for both isomers, so the signals reported are for the major (2*Z*,4*Z*)-isomer. For the minor isomer, only diagnostic vinylic C4-H could be unequivocally assigned (separately listed, *vide infra*). ^1^H-NMR (CDCl_3_) of (2*Z*,4*Z*)-isomer: *δ* 7.30–7.27 (m, 2H, Ar-H), 7.20–7.18 (m, 3H, Ar-H), 6.68 (ddd, 1H, *J* = 32.7; 11.7; 1.0 Hz), 6.34 (t, 1H, *J* = 11.2 Hz), 5.80 (dt, 1H, *J* = 10.7; 7.8 Hz), 3.74 (s, 3H, OCH_3_), 3.24 (s, 3H, CH_3_), 2.73 (t, 2H, *J* = 7.8 Hz), 2.55 (q, 2H, *J* = 7.8 Hz). Minor (2*Z*,4*E*)-isomer (CDCl_3_): *δ* 6.04 ppm (1H, C4-H, *J* = 14.7; 7.3 Hz). ^19^F-NMR (CDCl_3_): *δ* −124.2 (d, ^3^*J*_HF_ = 33.6 Hz, (4*Z*)-isomer), −125.7 (d, ^3^*J*_HF_ = 30.5 Hz, (4*E*)-isomer). HRMS (ESI) calcd for C_15_H_19_FNO_2_ [M+H]^+^ 264.1394, found 264.1407.

### 3.4. Isomerization of the (2Z,4E/Z)-Isomer Mixture of **6a–d** to the (2Z,4E)-Isomer

*General experimental procedure.* To a stirred solution of the (2*Z*,4*E/Z*)-isomer mixture **6** in dry CHCl_3_ was added I_2_ (7–10 mol %) and the mixture was stirred at rt. The reaction was monitored by ^19^F-NMR and when only one isomer was observed, the reaction mixture was diluted with EtOAc (30 mL). The mixture was washed with water, saturated aq. Na_2_S_2_O_3_ (2 × 10 mL), and dried over Na_2_SO_4_. The organic layer was concentrated under reduced pressure to afford the desired (2*Z*,4*E*)-isomer. HMQC data were obtained for (2*Z*,4*E*)-**6c** and (2*Z*,4*E*)-**6d**, and where unequivocally assigned, through-bond C–F couplings are reported as ^1^*J*_CF_, ^2^*J*_CF_, *etc*. Due to the close structural similarity, these couplings are also reported for (2*Z*,4*E*)-**6a** and (2*Z*,4*E*)-**6b**, where possible.

*(2Z,4E)-2-Fluoro-N-methoxy-N-methyl-5-(naphthalene-2-yl)penta-2,4-dienamide (2Z,4E)-***6a**. Isomerization was performed with (2*Z*,4*E*/*Z*)-**6a** (16.0 mg, 0.056 mmol) in CHCl_3_ (8.0 mL) using I_2_ (1.2 mg, 4.7 × 10^−3^ mmol, 8.4 mol %), in a reaction time of 3 h, to yield isomer (2*Z*,4*E*)-**6a** as a white solid (12.0 mg, 75%). R_f_ (SiO_2_, 30% EtOAc in hexanes): 0.49. ^1^H-NMR (CDCl_3_): *δ* 7.83–7.80 (m, 4H, Ar-H), 7.69 (dd, 1H, Ar-H, *J* = 8.3; 1.5 Hz), 7.50–7.46 (m, 2H, Ar-H), 7.21 (dd, 1H, *J* = 15.8; 11.3 Hz), 6.97 (d, 1H, *J* = 15.8 Hz), 6.72 (dd, 1H, *J* = 32.4; 11.3 Hz), 3.80 (s, 3H, OCH_3_), 3.29 (s, 3H, CH_3_). ^13^C-NMR (CDCl_3_): *δ* 162.8 (d, ^2^*J*_CF_ = 26.5 Hz), 150.2 (d, ^1^*J*_CF _ = 275.6 Hz), 137.9 (d, ^4^*J*_CF _ = 4.5 Hz), 134.1 (d, *J* = 1.9 Hz), 133.7, 128.7, 128.4, 128.0 (d, *J* = 1.8 Hz), 127.9, 126.8, 126.7, 123.6, 119.6 (d, ^3^*J*_CF_ = 3.7 Hz)_,_118.2 (d, ^2^*J*_CF_ = 9.2 Hz), 62.1 (d, ^5^*J*_CF_ = 2.8 Hz), 34.3. ^19^F-NMR (CDCl_3_): *δ* −123.4 (d, ^3^*J*_HF_ = 30.5 Hz).

*(2Z,4E)-2-Fluoro-N-methoxy-N-methyl-5-(4-methoxyphenyl)penta-2,4-dienamide (2Z,4E)-***6b**. Isomerization was performed with (2*Z*,4*E/Z*)-**6b** (14.0 mg, 0.053 mmol) in CHCl_3_ (8.0 mL) using I_2_ (1.3 mg, 5.1 × 10^−3^ mmol, 10 mol %), in a reaction time of 3 h, to yield isomer (2*Z*,4*E*)-**6b** as a pale yellow solid (12.0 mg, 86%). R_f_ (SiO_2_, 30% EtOAc in hexanes): 0.35. ^1^H-NMR (CDCl_3_): *δ* 7.42 (d, 2H, Ar-H, *J* = 7.3 Hz), 6.95 (dd, 1H, *J* = 15.6; 11.2 Hz), 6.88 (d, 2H, Ar-H, *J* = 6.8 Hz), 6.75 (d, 1H, *J* = 15.6 Hz), 6.65 (dd, 1H, *J* = 32.2; 11.2 Hz), 3.83 (s, 3H, OCH_3_), 3.77 (s, 3H, OCH_3_), 3.26 (s, 3H, CH_3_). ^13^C-NMR (CDCl_3_): *δ* 162.9 (d, ^2^*J*_CF_ = 26.8 Hz), 160.4, 149.5 (d, ^1^*J*_CF_ = 272.8 Hz), 137.5 (d, ^4^*J*_CF_ = 4.3 Hz), 129.5, 128.7, 118.7 (d, ^2^*J*_CF_ = 9.1 Hz), 117.2, 114.5, 62.1, 55.6, 34.3. ^19^F-NMR (CDCl_3_): *δ* –125.2 (d, ^3^*J*_HF_ = 33.6 Hz).

*(2Z,4E)-2-Fluoro-N-methoxy-N-methyl-5-(4-nitrophenyl)penta-2,4-dienamide (2Z,4E)-***6c**. Isomerization was performed with (2*Z*,4*E/Z*)-**6c** (4.5 mg, 0.017 mmol) in CHCl_3_ (1.6 mL) using I_2_ (0.3 mg, 1.2 × 10^−3^ mmol, 7 mol %), in a reaction time of 1.5 h, to yield isomer (2*Z*,4*E*)-**6c** as a yellow solid (4.0 mg, 89%). R_f_ (SiO_2_, 30% EtOAc in hexanes): 0.47. ^1^H-NMR (CDCl_3_): *δ* 8.21 (d, Ar-H, 2H, *J* = 8.8 Hz), 7.60 (d, Ar-H,2H, *J* = 8.8 Hz), 7.23 (dd, 1H, *J* = 15.6; 11.2 Hz), 6.83 (d, 1H, *J* = 15.6 Hz), 6.65 (dd, 1H, *J* = 31.7; 11.2 Hz), 3.79 (s, 3H, OCH_3_), 3.28 (s, 3H, CH_3_). ^13^C-NMR (CDCl_3_): *δ* 162.3 (d, C= O, ^2^*J*_CF_ = 26.6 Hz), 151.8 (d, C-F, ^1^*J*_CF _ = 280.1 Hz), 147.6, 142.9, 134.7 (d, ^4^*J*_CF_ = 4.6 Hz), 127.6, 124.4, 123.6 (d, ^3^*J*_CF_ = 3.7 Hz), 116.8 (d, ^2^*J*_CF_* = * 9.2 Hz), 62.2 (d, ^5^*J*_CF_ = 2.3 Hz), 34.2. ^19^F-NMR (CDCl_3_): *δ* −119.6 (d, ^3^*J*_HF_ = 30.5 Hz).

*(2Z,4E)-2-Fluoro-N-methoxy-N-methyl-5-(thiophen-2-yl)penta-2,4-dienamide (2Z,4E)-***6d**. Isomerization was performed with (2*Z*,4*E/Z*)-**6d** (12.0 mg, 0.050 mmol) in CHCl_3_ (7.5 mL) using I_2_ (1.2 mg, 4.7 × 10^−3^ mmol, 10 mol %), in an overnight reaction, to yield isomer (2*Z*,4*E*)-**6d** as an off-white solid (11.0 mg, 92%). R_f_ (SiO_2_, 30% EtOAc in hexanes): 0.34. ^1^H-NMR (CDCl_3_): *δ* 7.26 (d, 1H, Ar-H, *J* = 5.4 Hz), 7.09 (d, 1H, Ar-H, *J* = 3.4 Hz), 7.00 (dd, 1H, Ar-H, *J* = 5.1; 3.7 Hz), 6.92 (d, 1H, *J* = 15.6 Hz), 6.86 (dd, 1H, *J* = 15.6; 10.7 Hz), 6.61 (dd, 1H, *J* = 32.2; 10.7 Hz), 3.77 (s, 3H, OCH_3_), 3.26 (s, 3H, CH_3_). ^13^C-NMR (CDCl_3_): *δ* 162.7 (d, ^2^*J*_CF_ = 26.5 Hz), 150.1 (d, ^1^*J*_CF _ = 275.5 Hz), 142.1 (d, *J* = 2.3 Hz), 130.4 (d, ^4^*J*_CF_ = 5.0 Hz), 128.1 (d, *J* = 1.8 Hz), 128.0, 126.5 (d, *J* = 1.4 Hz), 118.9 (d, ^3^*J*_CF _*= * 3.2 Hz), 117.9 (d, ^2^*J*_CF_* = * 9.6 Hz), 62.1 (d, *J* = 2.7 Hz), 34.3. ^19^F-NMR (CDCl_3_): *δ* −123.7 (d, ^3^*J*_HF_ = 30.5 Hz).

## 4. Conclusions

In conclusion, we have developed a highly modular approach to (2*Z*,4*E*)-2-fluoro-2,4-dienamides. This was achieved via two sequential Julia-Kocienski olefinations. In the first olefination, a *Z*-α-fluorovinyl Weinreb amide unit, with a benzothiazolylsulfanyl substituent at the allylic position, was assembled. For this, two *key building blocks*, a known fluorinated Julia-Kocienski reagent with a Weinreb amide moiety (**1**) and 2-(benzo[*d*]thiazol-2-ylthio)acetaldehyde (**2**), a precursor to the second Julia-Kocienski reagent, were reacted. Condensation proceeded with high *Z*-stereoselectivity and in a good 76% yield. Oxidation of the sulfide to the sulfone furnished the “second-generation” Julia-Kocienski olefination reagent, which underwent reactions with aldehydes to furnish the dienamides in 50%–74% yields. The second set of olefinations proceeded under DBU-mediated conditions and with *Z*-stereoselectivity. Isomeric (2*Z*,4*E*/*Z*)-mixtures underwent iodine-mediated isomerization to a single (2*Z*,4*E*)-dienamide isomer. Although the method was performed with a Weinreb amide moiety, it is potentially applicable to other amides as well. Moreover, due to versatile chemistry of the Weinreb amide moiety, the (2*Z*,4*E*)-2-fluoro Weinreb dienamides are potentially useful synthetic building blocks, which can undergo a variety of conversions leading to more complex molecules. In this context, the present method offers a straightforward access to synthetically valuable entities that are not otherwise easily prepared by traditional synthetic approaches to Weinreb amides.

## Supplementary Materials

Supplementary materials can be accessed at: http://www.mdpi.com/1420-3049/19/4/4418/s1.

## References

[1] Nájera C., Yus M., Rahman A. (2000). Natural products with polyene amide structures. Bioactive Natural Products (Part B). Studies in Natural Products Chemistry.

[2] Tsuji N., Kobayashi M., Nagashima K. (1976). A new antifungal antibiotic, trichostatin. J. Antibiot..

[3] Yoshida M., Kijima M., Akita M., Beppu T. (1990). Potent and specific inhibition of mammalian histone deacetylase both *in vivo* and *in vitro* by trichostatin A. J. Biol. Chem..

[4] Drummond D.C., Noble C.O., Kirpotin D.B., Guo Z., Scott G.K., Benz C.C. (2005). Clinical development of histone deacetylase inhibitors as anticancer agents. Annu. Rev. Pharmacol. Toxicol..

[5] Jacobson M. (1949). The structure of pellitorine. J. Am. Chem. Soc..

[6] Ee G.C.L., Lim C.M., Rahmani M., Shaari K., Bong C.F.J. (2010). Pellitorine, a potential anti-cancer lead compound against HL60 and MTC–7 cell lines and microbial transformation of piperine from *Piper nigrum*. Molecules.

[7] Bezerra D.P., Pessoa C., de Moraes M.O., Saker-Neto N., Silveira E.R., Costa-Lotufo L.V. (2013). Overview of the therapeutic potential of piplartine (piperlongumine). Eur. J. Pharm. Sci..

[8] Bezerra D.P., Pessoa C., de Moraes M.O., de Alencar N.M.N., Mesquita R.O., Lima M.W., Alves A.P.N.N., Pessoa O.D.L., Chaves J.H., Silveira E.R. (2008). *In vivo* growth inhibition of sarcoma 180 by piperlonguminine, an alkaloid amide from the *Piper* species. J. Appl. Toxicol..

[9] Lee W., Yoo H., Ku S.-K., Kim J.A., Bae J.-S. (2013). Anticoagulant activities of piperlonguminine *in vitro* and *in vivo*. BMP Rep..

[10] Kim K.-S., Kim J.A., Eom S.-Y., Lee S.H., Min K.R., Kim Y. (2006). Inhibitory effect of piperlonguminine on melanin production in melanoma B16 cell line by downregulation of tyrosinase expression. Pigment Cell Res..

[11] Rodrigues Silva D., Baroni S., Svidzinski A.E., Bersani-Amado C.A., Cortez D.A.G. (2008). Anti-inflammatory activity of the extract, fractions and amides from the leaves of *Piper ovatum* Vahl (Piperaceae). J. Ethnopharmacol..

[12] Makapugay H.C., Soejarto D.D., Kinghorn A.D., Bordas E. (1983). Piperovatine, the tongue-numbing principle of *Ottonia frutescens*. J. Ethnopharmacol..

[13] Charrier C., Roche J., Gesson J.-P., Bertrand P. (2009). Biological activities of substituted trichostatic acid derivatives. J. Chem. Sci..

[14] Welch J.T. (1991). Selective Fluorination in Organic and Bioorganic Chemistry.

[15] Kirsch P. (2004). Modern Fluoroorganic Chemistry. Synthesis, Reactivity, Applications.

[16] Bégué J.-P., Bonnet-Delpon D. (2008). Bioorganic and Medicinal Chemistry of Fluorine.

[17] Smart B.E. (2001). Fluorine substituent effects (on bioactivity). J. Fluorine Chem..

[18] Lemal D.M. (2004). Perspective on fluorocarbon chemistry. J. Org. Chem..

[19] O’Hagan D. (2008). Understanding organofluorine chemistry. An introduction to the C–F bond. Chem. Soc. Rev..

[20] Blakemore P.R. (2002). The modified Julia olefination: alkene synthesis *via* the condensation of metallated heteroarylalkylsulfones with carbonyl compounds. J. Chem. Soc. Perkin Trans. I.

[21] Plesniak K., Zarecki A., Wicha J. (2007). The Smiles rearrangement and the Julia-Kocienski olefination reaction. Top. Curr. Chem..

[22] Aïssa C. (2009). Mechanistic manifold and new developments of the Julia-Kocienski reaction. Eur. J. Org. Chem..

[23] Blakemore P.R., Knochel P., Molander G.A. (2014). Olefination of carbonyl compounds by main-group element mediators. Comprehensive Organic Synthesis.

[24] Zajc B., Kumar R. (2010). Synthesis of fluoroolefins via Julia-Kocienski olefination. Synthesis.

[25] Landelle G., Bergeron M., Turcotte-Savard M.-O., Paquin J.-P. (2011). Synthetic approaches to monofluoroalkenes. Chem. Soc. Rev..

[26] Yanai H., Taguchi T. (2011). Synthetic methods for fluorinated olefins. Eur. J. Org. Chem..

[27] Ghosh A.K., Zajc B. (2006). High-yield synthesis of fluorinated benzothiazolyl sulfones: General synthons for fluoro-Julia olefinations. Org. Lett..

[28] Zajc B., Kake S. (2006). Exceptionally mild, high-yield synthesis of α-fluoro acrylates. Org. Lett..

[29] He M., Ghosh A.K., Zajc B. (2008). Julia olefination as a general route to phenyl (α-fluoro)vinyl sulfones. Synlett.

[30] del Solar M., Ghosh A.K., Zajc B. (2008). Fluoro-Julia olefination as a mild, high-yielding route to α-fluoro acrylonitriles. J. Org. Chem..

[31] Ghosh A.K., Banerjee S., Sinha S., Kang S.B., Zajc B. (2009). α-Fluorovinyl Weinreb amides and α-fluoroenones from a common fluorinated building block. J. Org. Chem..

[32] Ghosh A.K., Zajc B. (2009). Fluorinated 1-phenyl-1*H*-tetrazol-5-yl sulfonederivatives as general reagents for fluoroalkylidene synthesis. J. Org. Chem..

[33] Kumar R., Pradhan P., Zajc B. (2011). Facile synthesis of 4-vinyl- and 4-fluorovinyl-1,2,3-triazoles via bifunctional “*click-olefination*” reagents. Chem. Commun..

[34] Mandal S.K., Ghosh A.K., Kumar R., Zajc B. (2012). Expedient synthesis of α-substituted fluoroethenes. Org. Biomol. Chem..

[35] Kumar R., Zajc B. (2012). Stereoselective synthesis of conjugated fluoro enynes. J. Org. Chem..

[36] Chevrie D., Lequeux T., Demoute J.P., Pazenok S. (2003). A convenient one-step synthesis of fluoroethylidene derivatives. Tetrahedron Lett..

[37] Pfund E., Lebargy C., Rouden J., Lequeux T. (2007). Modified Julia fluoroolefination: Selective preparation of fluoroalkenoates. J. Org. Chem..

[38] Alonso D.A., Fuensanta M., Gómez-Bengoa E., Nájera C. (2008). Highly efficient and stereoselective Julia-Kocienski protocol for the synthesis of α-fluoro-α,β-unsaturated esters and Weinreb amides employing 3,5-bis(trifluoromethyl)phenyl (BTFP) sulfones. Adv. Synth. Catal..

[39] Calata C., Catel J.-M., Pfund E., Lequeux T. (2009). Scope and limitations of the Julia-Kocienski reaction with fluorinated sulfonylesters. Tetrahedron.

[40] Calata C., Pfund E., Lequeux T. (2009). Toward the synthesis of benzothiazolyl fluoroaminosulfones. J. Org. Chem..

[41] Zhao Y., Huang W., Zhu L., Hu J. (2010). Difluoromethyl 2-pyridyl sulfone: A new *gem*-difluoroolefination reagent for aldehydes and ketones. Org. Lett..

[42] Prakash G.K.S., Shakhmin A., Zibinsky M., Ledneczki I., Chacko S., Olah G.A. (2010). Synthesis of monofluoroalkenes via Julia-Kocienski reaction. J. Fluorine Chem..

[43] Allendörfer N., Es-Sayed M., Nieger M., Bräse S. (2010). Novel aromatic fluoroolefins via fluoro-Julia-Kocienski olefination. Synthesis.

[44] Calata C., Pfund E., Lequeux T. (2011). Convergent synthesis of functionalized fluoroallylamines by the Julia-Kocienski reaction. Tetrahedron.

[45] Jacobsen C.B., Nielsen M., Worgull D., Zweifel T., Fisker E., Jørgensen K.A. (2011). Asymmetric organocatalytic monofluorovinylations. J. Am. Chem. Soc..

[46] Larnaud F., Malassis J., Pfund E., Linclau B., Lequeux T. (2013). Ready synthetic access to enantiopure allylic α_(F)_-branched fluoroalkenes. Org. Lett..

[47] Singh G., Kumar R., Swett J., Zajc B. (2013). Modular Synthesis of *N*-Vinyl Benzotriazoles. Org. Lett..

[48] Nahm S., Weinreb S.M. (1981). *N*-Methoxy-*N*-methylamides as effective acylating agents. Tetrahedron Lett..

[49] Khlestkin V.K., Mazhukin D.G. (2003). Recent advances in the application of N,O-dialkylhydroxylamines in organic chemistry. Curr. Org. Chem..

[50] Balasubramaniam S., Aidhen I.S. (2008). The growing synthetic utility of the Weinreb amide. Synthesis.

[51] Pace V., Castoldi L., Holzer W. (2013). Synthesis of *α,β*-unsaturated *α’*-haloketones through the chemoselective addition of halomethyllithiums to Weinreb amides. J. Org. Chem..

[52] Giesbrecht H.E., Knight B.J., Tanguileg N.R., Emerson C.R., Blakemore P.R. (2010). Stereoselective synthesis of *Z*-configured αβ-unsaturated macrocyclic lactones and diolides by intramolecular Julia-Kocienski olefination. Synlett.

[53] Gaukroger K., Hadfield J.A., Hepworth L.A., Lawrence N.J., McGown A.T. (2001). Novel syntheses of *cis* and *trans* isomers of combretastatin A-4. J. Org. Chem..

[54] Manjunath B.N., Sane N.P., Aidhen I.S. (2006). New reagent for convenient access to the α,β-unsaturated *N*-methoxy-*N*-methyl-amide functionality by a synthesis based on the Julia olefination protocol. Eur. J. Org. Chem..

