# Supplementary material for: Synthesis of Regiospecifically Fluorinated Conjugated Dienamides
Source: Molecules. 2014 Apr 10;19(4):4418–32. doi: 10.3390/molecules19044418 (PMC4440806; doi:10.3390/molecules19044418)

Supporting Information

Synthesis of Regiospecifically Fluorinated 1,3-Dienamides

Mohammad Chowdhury, Samir K. Mandal, Shaibal Banerjee and Barbara Zajc *

Department of Chemistry, The City College and The City University of New York, 160 Convent Avenue, NY 10031, USA

TABLE OF CONTENTS

Information	Page
^{1}H NMR of 2-(benzo[*d*]thiazol-2-ylsulfonyl)-2-fluoro-*N*-methoxy-*N*-methylacetamide (**1**)	**S2**
^{19}F NMR of 2-(benzo[*d*]thiazol-2-ylsulfonyl)-2-fluoro-*N*-methoxy-*N*-methylacetamide (**1**)	**S3**
^{1}H NMR of 2-(2,2-dimethoxyethylthio)benzo[*d*]thiazole (**3**)	**S4**
^{13}C NMR of 2-(2,2-dimethoxyethylthio)benzo[*d*]thiazole (**3**)	**S5**
^{1}H NMR of 2-(benzo[*d*]thiazol-2-ylthio)acetaldehyde (**2**)	**S6**
^{1}H NMR of (*Z*)-4-(benzo[*d*]thiazol-2-ylthio)-2-fluoro-*N*-methoxy-*N*-methylbut-2-enamide (**4**)	**S7**
^{13}C NMR of (*Z*)-4-(benzo[*d*]thiazol-2-ylthio)-2-fluoro-*N*-methoxy-*N*-methylbut-2-enamide (**4**)	**S8**
^{19}F NMR of (*Z*)-4-(benzo[*d*]thiazol-2-ylthio)-2-fluoro-*N*-methoxy-*N*-methylbut-2-enamide (**4**)	**S9**
^{1}H NMR of (*Z*)-4-(benzo[*d*]thiazol-2-ylsulfonyl)-2-fluoro-*N*-methoxy-*N*-methylbut-2-enamide (**5**)	**S10**
^{13}C NMR of (*Z*)-4-(benzo[*d*]thiazol-2-ylsulfonyl)-2-fluoro-*N*-methoxy-*N*-methylbut-2-enamide (**5**)	**S11**
^{19}F NMR of (*Z*)-4-(benzo[*d*]thiazol-2-ylsulfonyl)-2-fluoro-*N*-methoxy-*N*-methylbut-2-enamide (**5**)	**S12**
^{1}H NMR of (2Z,4*E*/*Z*)-**6a**	**S13**
^{19}F NMR of (2Z,4*E*/*Z*)-**6a**	**S14**
^{1}H NMR of (2Z,4*E*/*Z*)-**6b**	**S15**
^{19}F NMR of (2Z,4*E*/*Z*)-**6b**	**S16**
^{1}H NMR of (2Z,4*E*/*Z*)-**6c**	**S17**
^{19}F NMR of (2Z,4*E*/*Z*)-**6c**	**S18**
^{1}H NMR of (2Z,4*E*/*Z*)-**6d**	**S19**
^{19}F NMR of (2Z,4*E*/*Z*)-**6d**	**S20**
^{1}H NMR of (2Z,4*E*/*Z*)-**6e**	**S21**
^{19}F NMR of (2Z,4*E*/*Z*)-**6e**	**S22**
^{1}H NMR of (2Z,4*E*)-**6a**	**S23**
^{13}C NMR of (2Z,4*E*)-**6a**	**S24**
^{19}F NMR of (2Z,4*E*)-**6a**	**S25**
^{1}H NMR of (2Z,4*E*)-**6b**	**S26**
^{13}C NMR of (2Z,4*E*)-**6b**	**S27**
^{19}F NMR of (2Z,4*E*)-**6b**	**S28**
^{1}H NMR of (2Z,4*E*)-**6c**	**S29**
^{13}C NMR of (2Z,4*E*)-**6c**	**S30**
^{19}F NMR of (2Z,4*E*)-**6c**	**S31**
^{1}H NMR of (2Z,4*E*)-**6d**	**S32**
^{13}C NMR of (2Z,4*E*)-**6d**	**S33**
^{19}F NMR of (2Z,4*E*)-**6d**	**S34**

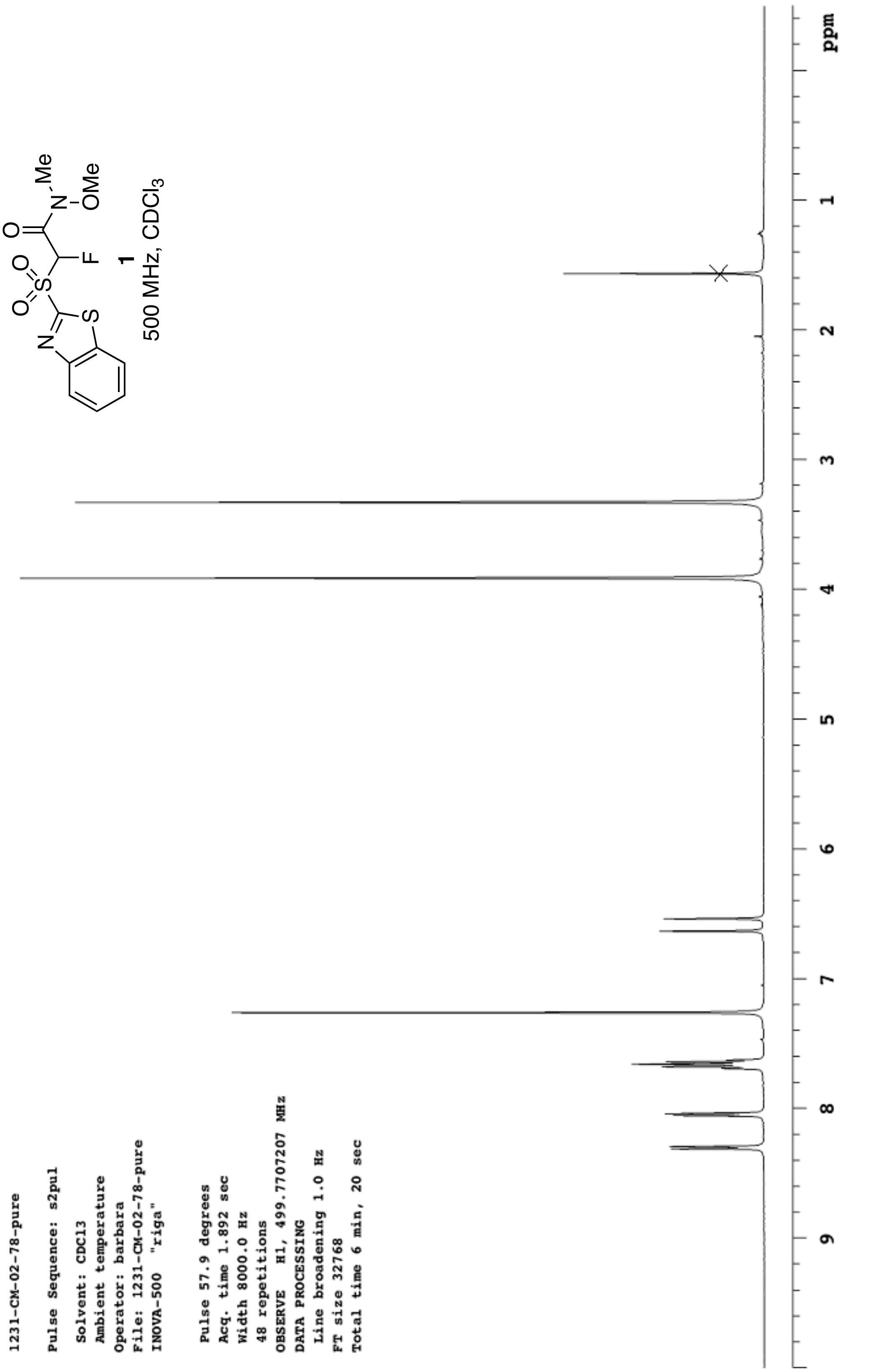
1231-CM-02-78-pure
Pulse Sequence: s2pul
Solvent: CDCl3
Ambient temperature
Operator: barbara
File: 1231-CM-02-78-pure
INOVA-500 "riga"
Pulse 57.9 degrees
Acq. time 1.892 sec
Width 8000.0 Hz
48 repetitions
OBSERVE H1, 499.7707207 MHz
DATA PROCESSING
Line broadening 1.0 Hz
FT size 32768
Total time 6 min, 20 sec
1
500 MHz, CDCl3
ppm
9 8 7 6 5 4 3 2 1

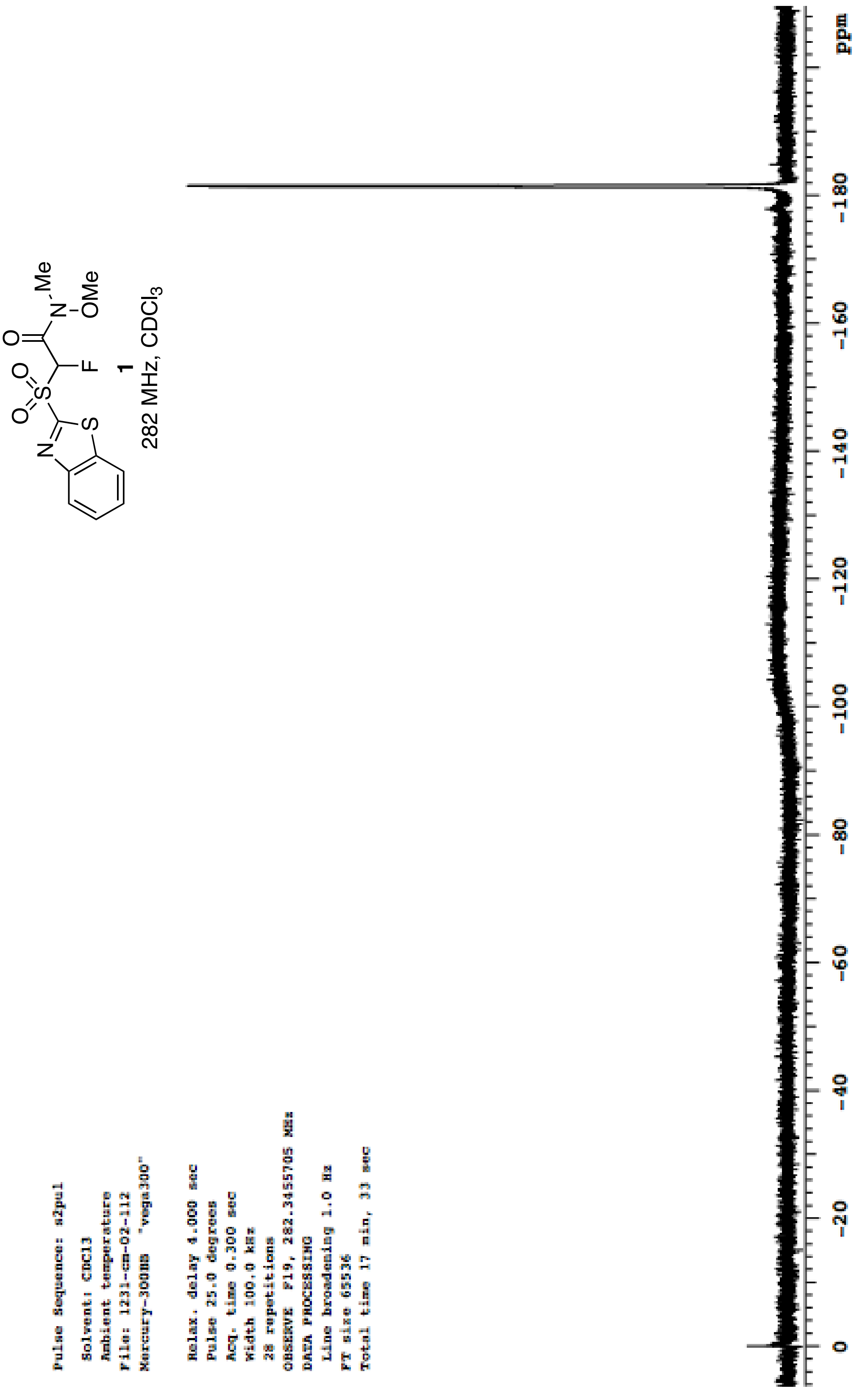
Me
N
OMe
O
S
O
F
N
S
1
282 MHz, CDCl3
Pulse Sequence: s2pul
Solvent: CDCl3
Ambient temperature
File: 1231-cm-02-112
Mercury-300BB "vega300"
Relax. delay 4.000 sec
Pulse 25.0 degrees
Acq. time 0.300 sec
Width 100.0 kHz
28 repetitions
OBSERVE F19, 282.3455705 MHz
DATA PROCESSING
Line broadening 1.0 Hz
FT size 65536
Total time 17 min, 33 sec
ppm
-180
-160
-140
-120
-100
-80
-60
-40
-20
0

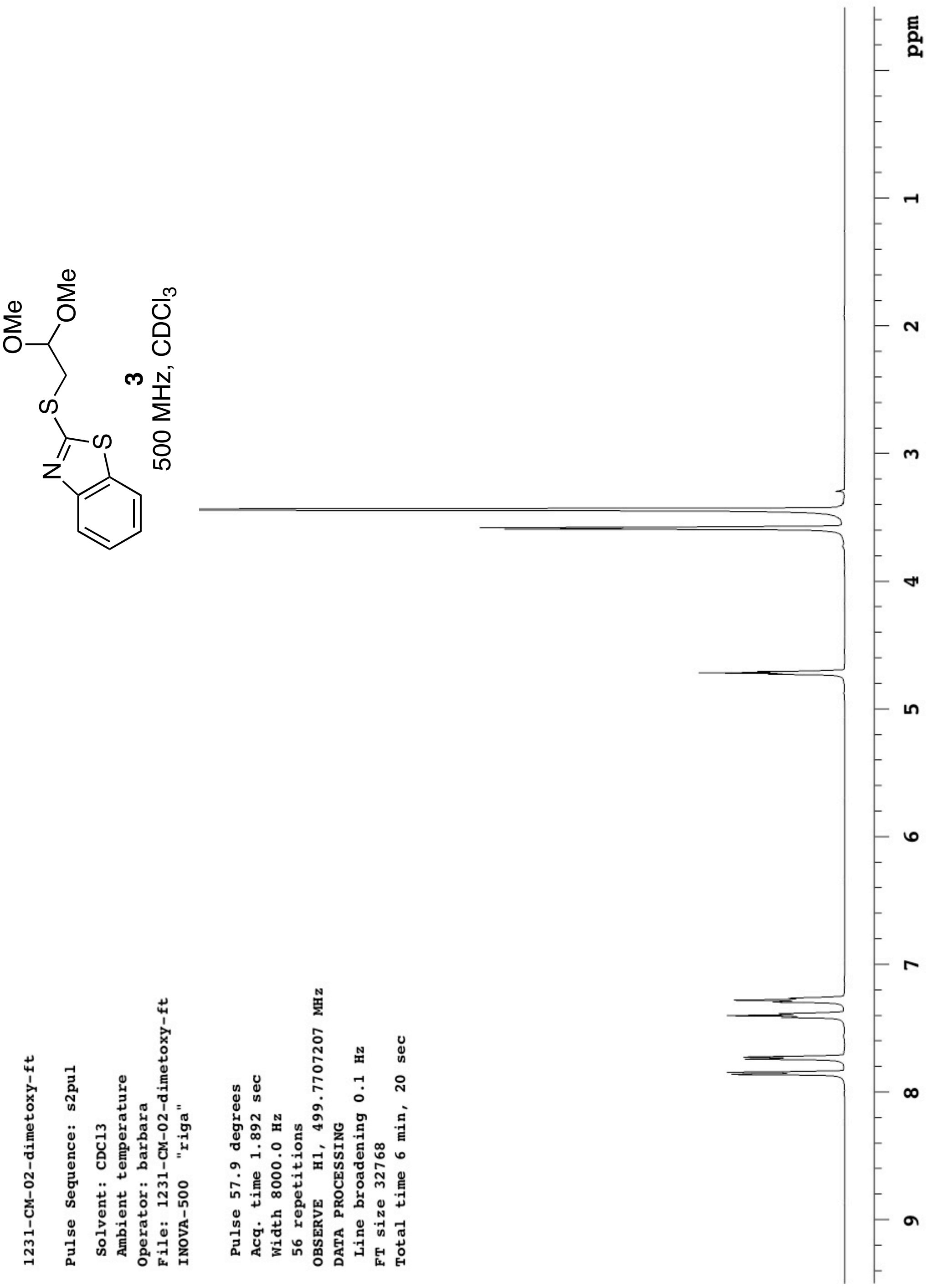
OMe
OMe
S
N
S
3
500 MHz, CDCl3
1231-CM-02-dimetoxy-ft
Pulse Sequence: s2pul
Solvent: CDCl3
Ambient temperature
Operator: barbara
File: 1231-CM-02-dimetoxy-ft
INOVA-500 "riga"
Pulse 57.9 degrees
Acq. time 1.892 sec
Width 8000.0 Hz
56 repetitions
OBSERVE H1, 499.7707207 MHz
DATA PROCESSING
Line broadening 0.1 Hz
FT size 32768
Total time 6 min, 20 sec
ppm
1
2
3
4
5
6
7
8
9

1231-CM-02-dimethoxy-sulfide-13C

Pulse Sequence: s2pul

Solvent: CDCl3
Temp. 25.0 C / 298.1 K
Operator: barbara
File: 1231-CM-02-dimethoxy-sulfide-13C
INOVA-500 "riga"

Relax. delay 4.000 sec
Pulse 52.1 degrees
Acq. time 1.300 sec
Width 29996.3 Hz
44 repetitions
OBSERVE C13, 125.6674328 MHz
DECOUPLE H1, 499.7732084 MHz
Power 42 dB
on during acquisition
WALTZ-16 modulated
DATA PROCESSING
Line broadening 0.0 Hz
FT size 131072
Total time 96 hr, 39 min, 38 sec

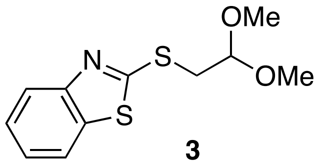

3
125 MHz, $CDCl_3$

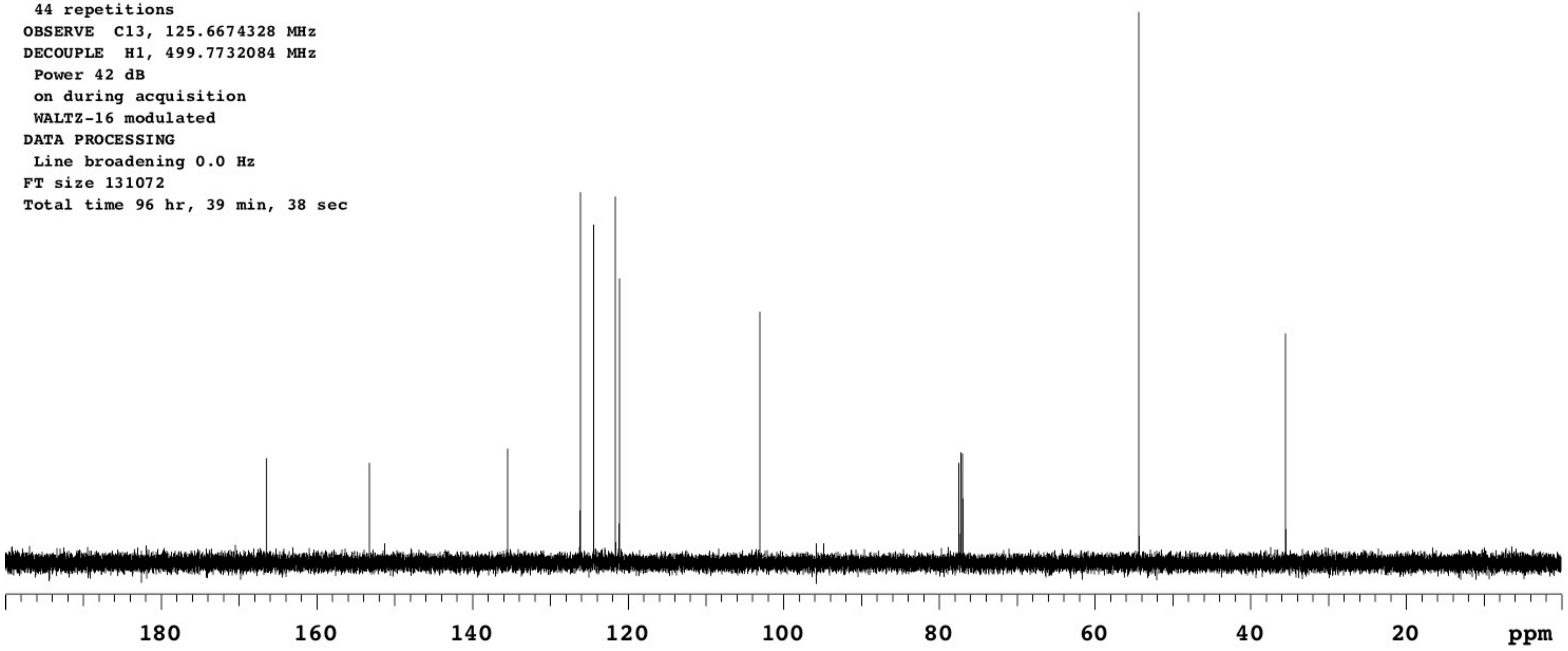

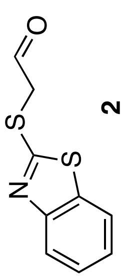

2
500 MHz, $CDCl_3$

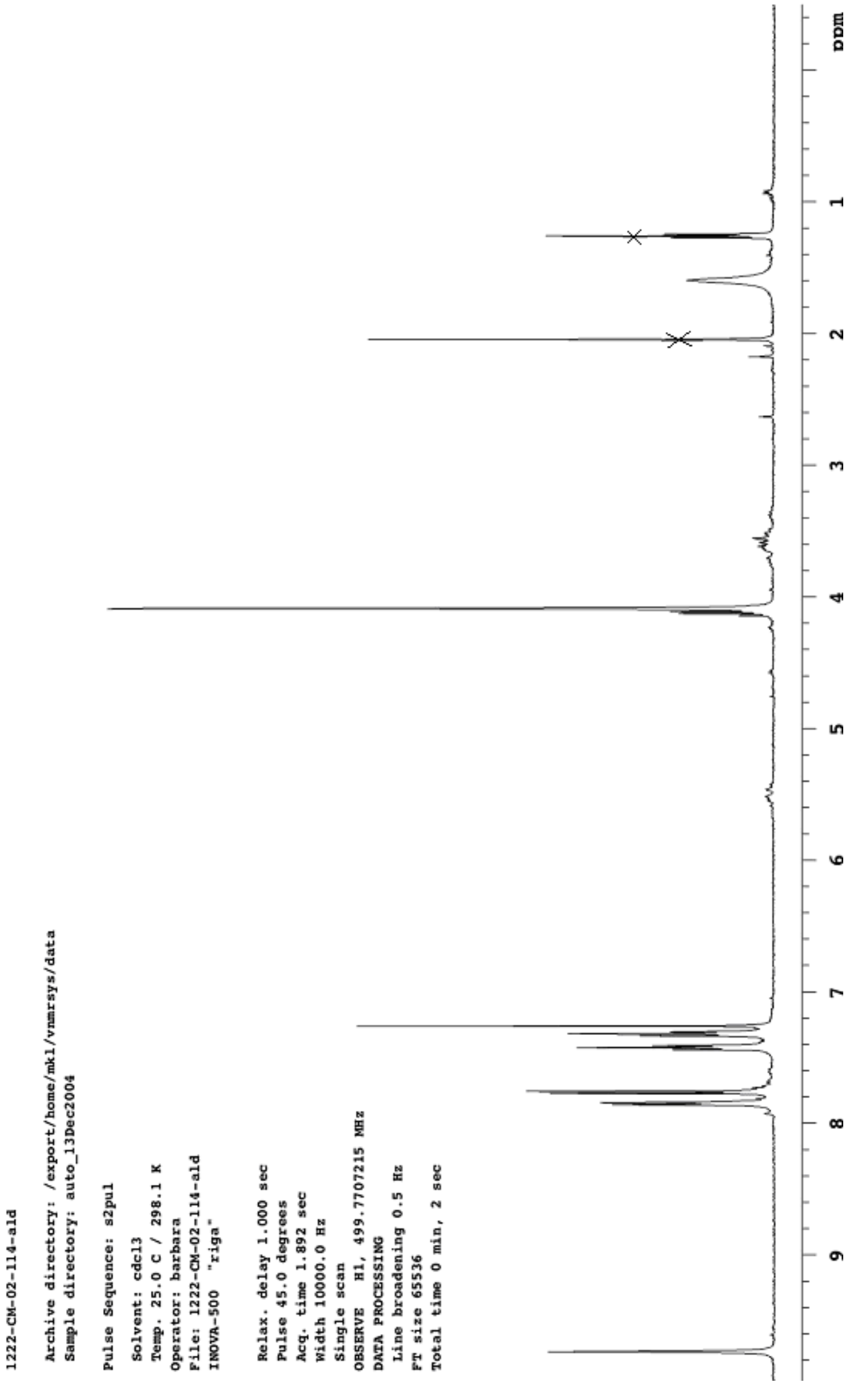

```
1231-cm-02-154-pure

Pulse Sequence: s2pul

 Solvent: CDCl3
 Ambient temperature
Operator: barbara
File: 1231-cm-02-154-pure
INOVA-500  "riga"

 Pulse 57.9 degrees
 Acq. time 1.892 sec
 Width 8000.0 Hz
 92 repetitions
OBSERVE   H1, 499.7707202 MHz
DATA PROCESSING
 Line broadening 0.1 Hz
FT size 32768
Total time 6 min, 20 sec
```

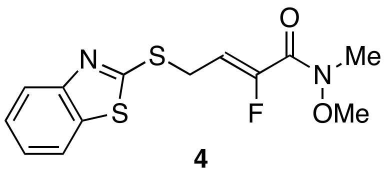

4

500 MHz, $CDCl_3$

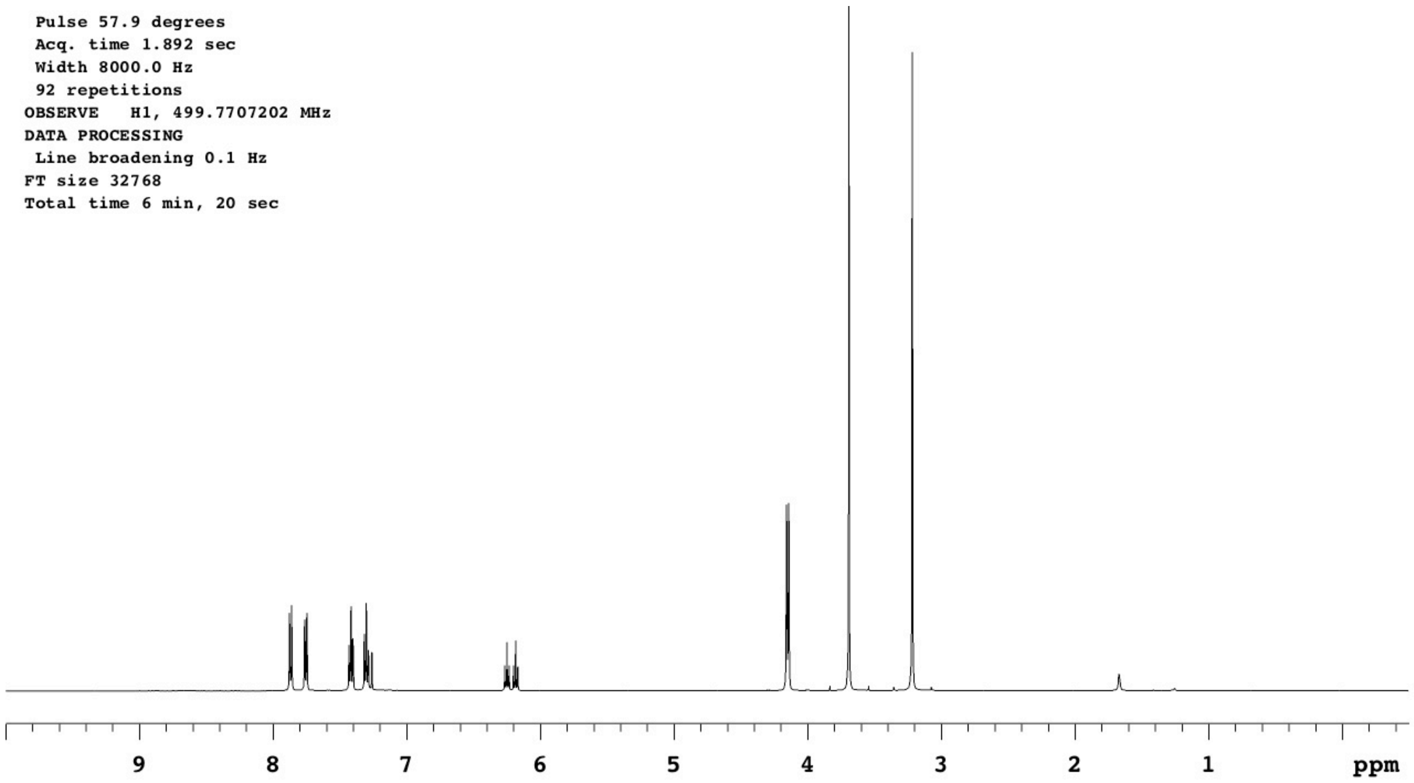

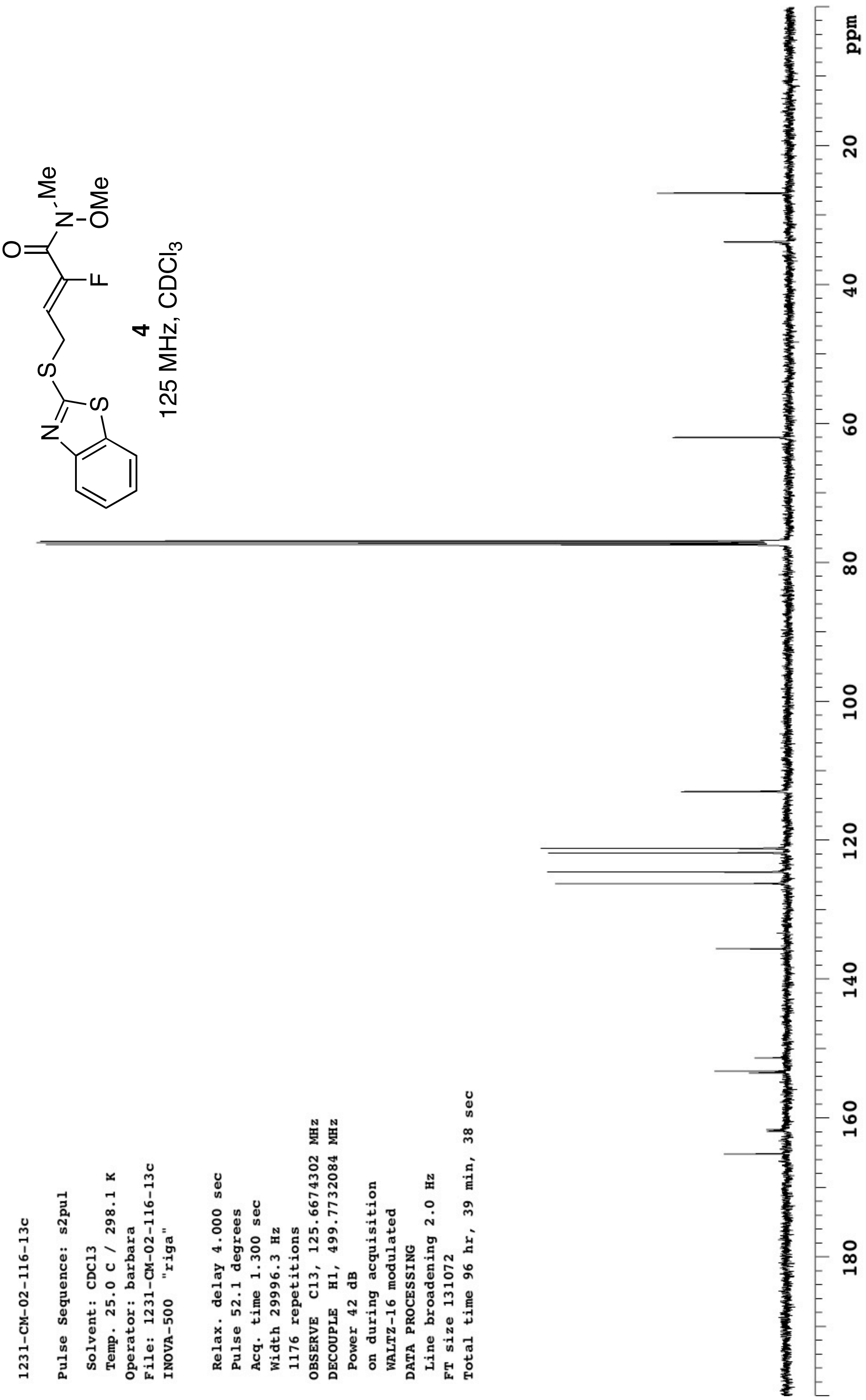
O
N
Me
OMe
F
S
N
S
4
125 MHz, CDCl3
1231-CM-02-116-13c
Pulse Sequence: s2pul
Solvent: CDCl3
Temp. 25.0 C / 298.1 K
Operator: barbara
File: 1231-CM-02-116-13c
INOVA-500 "riga"
Relax. delay 4.000 sec
Pulse 52.1 degrees
Acq. time 1.300 sec
Width 29996.3 Hz
1176 repetitions
OBSERVE C13, 125.6674302 MHz
DECOUPLE H1, 499.7732084 MHz
Power 42 dB
on during acquisition
WALTZ-16 modulated
DATA PROCESSING
Line broadening 2.0 Hz
FT size 131072
Total time 96 hr, 39 min, 38 sec
ppm
20
40
60
80
100
120
140
160
180

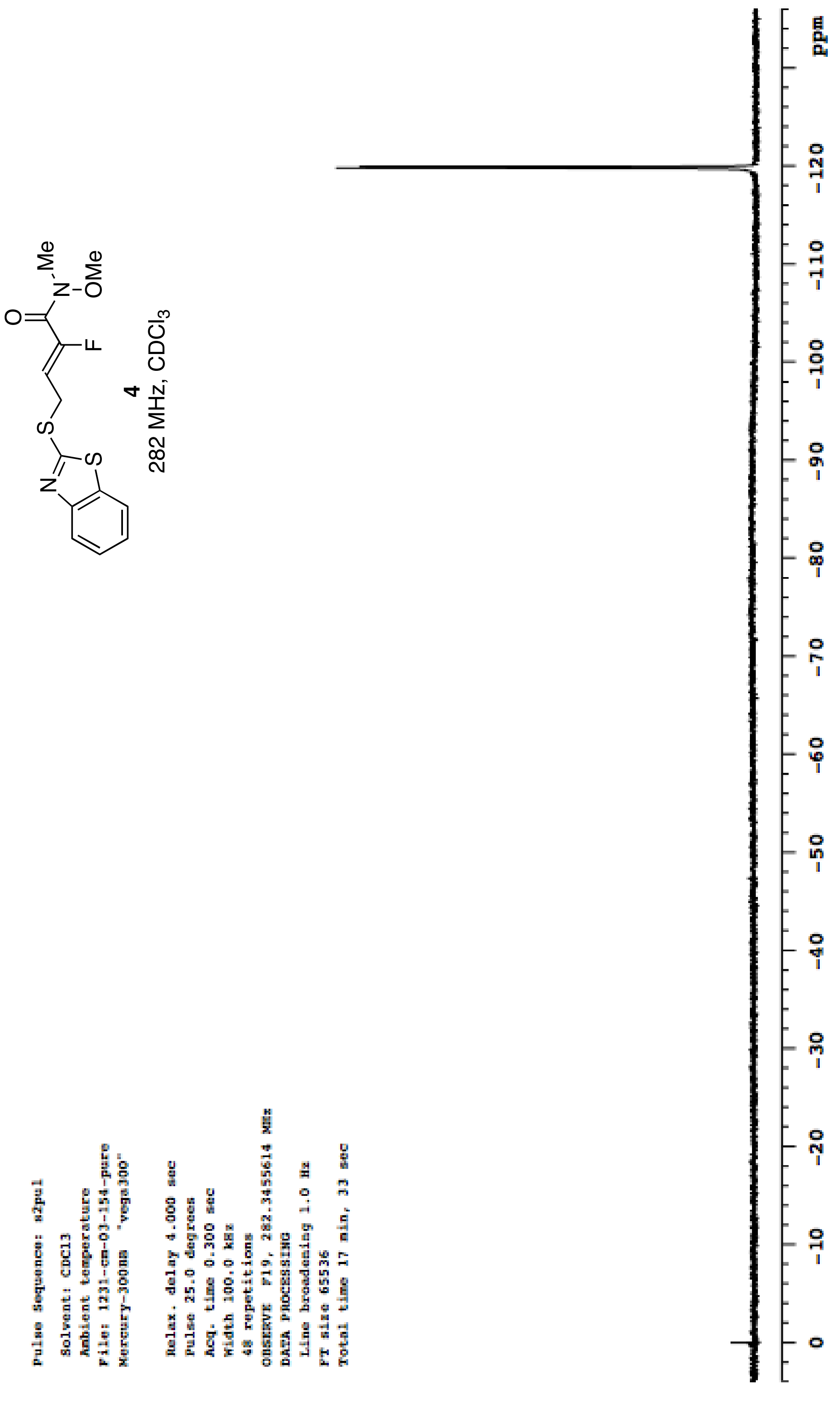
Pulse Sequence: s2pul
Solvent: CDCl3
Ambient temperature
File: 1231-cm-03-154-pure
Mercury-300BB "vega300"
Relax. delay 4.000 sec
Pulse 25.0 degrees
Acq. time 0.300 sec
Width 100.0 kHz
48 repetitions
OBSERVE F19, 282.3455614 MHz
DATA PROCESSING
Line broadening 1.0 Hz
FT size 65536
Total time 17 min, 33 sec
O
N
Me
OMe
F
S
N
S
4
282 MHz, $CDCl_3$
-120
-110
-100
-90
-80
-70
-60
-50
-40
-30
-20
-10
0
ppm

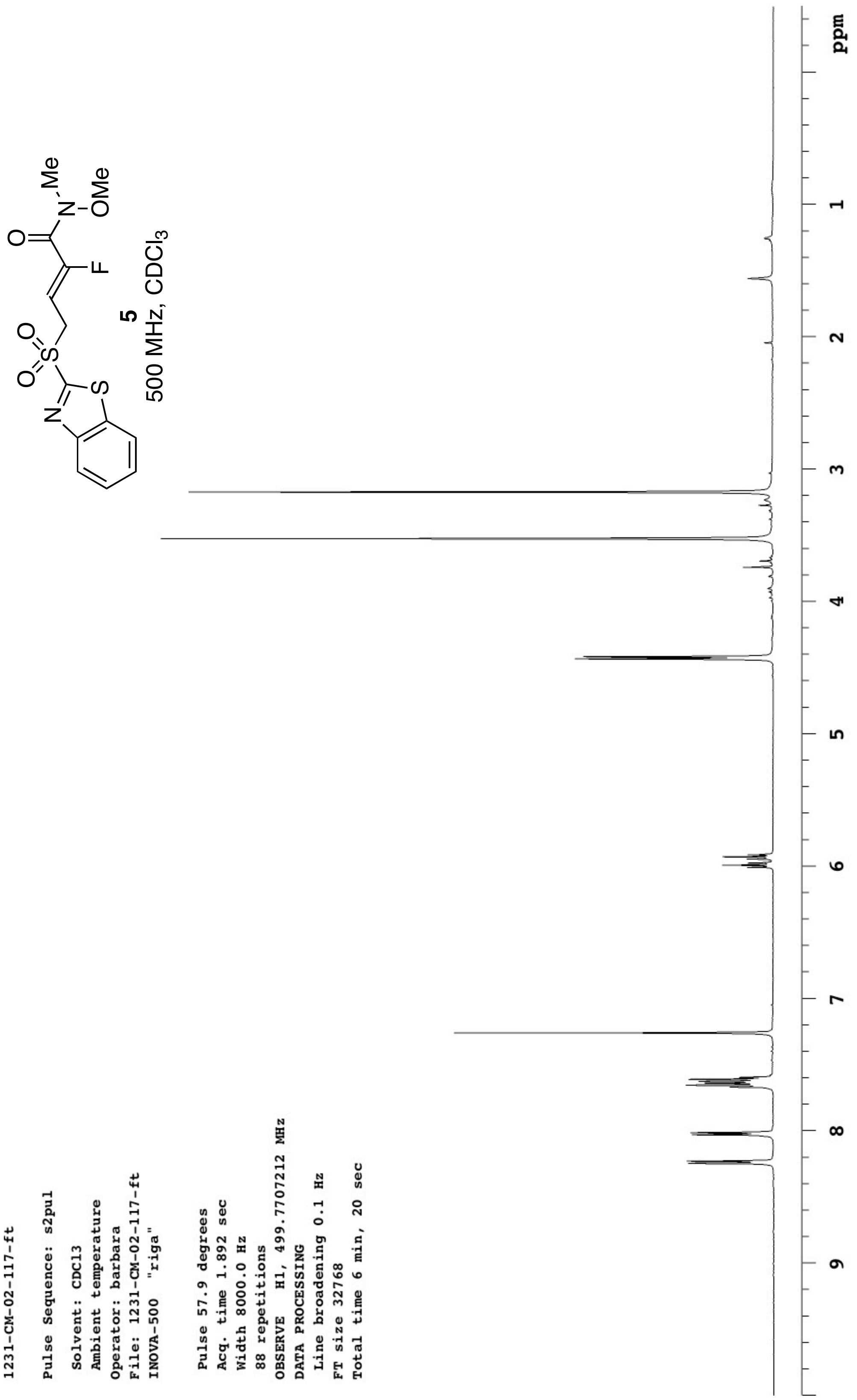
1231-CM-02-117-ft
Pulse Sequence: s2pul
Solvent: CDCl3
Ambient temperature
Operator: barbara
File: 1231-CM-02-117-ft
INOVA-500 "riga"
Pulse 57.9 degrees
Acq. time 1.892 sec
Width 8000.0 Hz
88 repetitions
OBSERVE H1, 499.7707212 MHz
DATA PROCESSING
Line broadening 0.1 Hz
FT size 32768
Total time 6 min, 20 sec
5
500 MHz, CDCl3
ppm

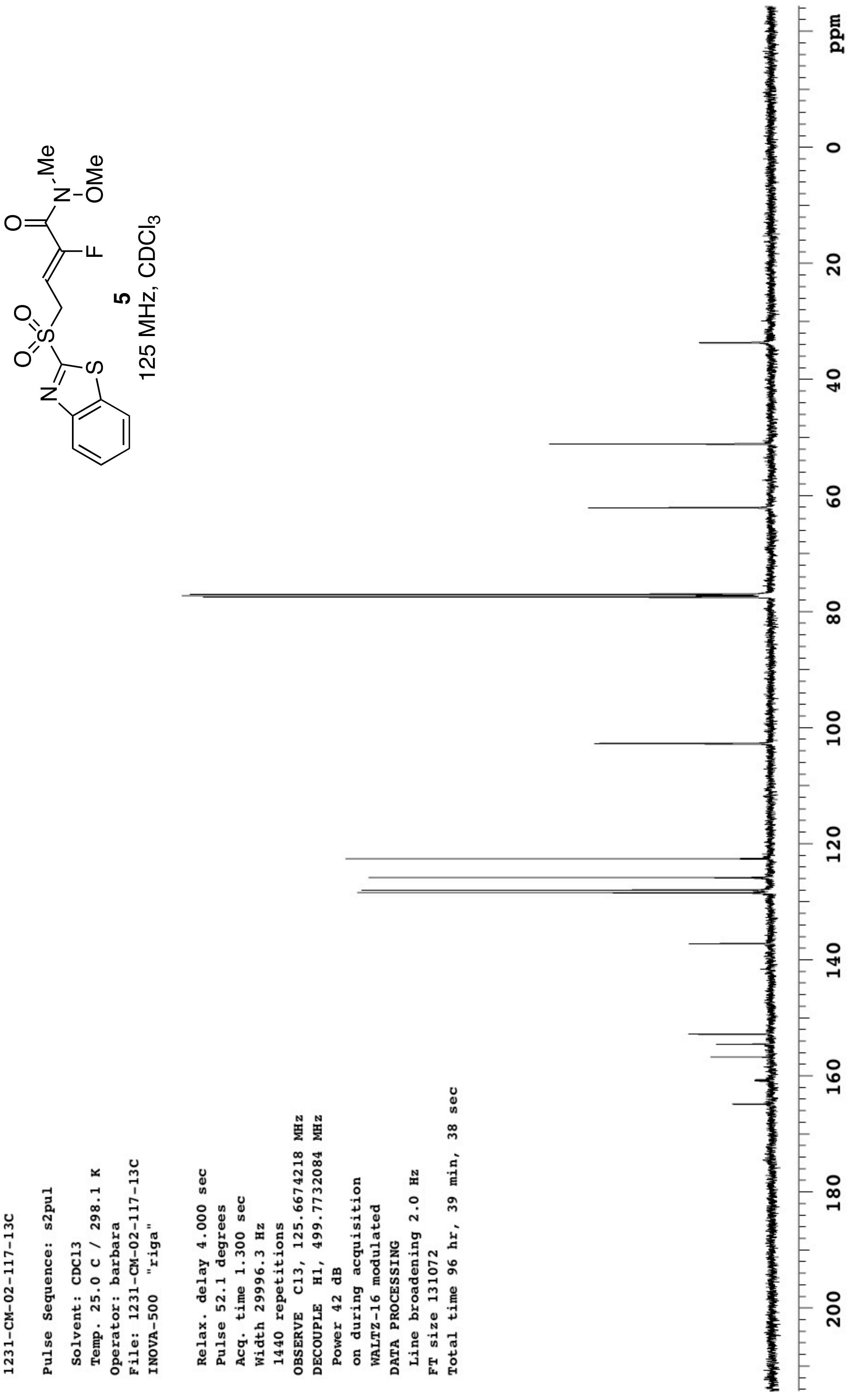
5
125 MHz, $CDCl_3$
1231-CM-02-117-13C
Pulse Sequence: s2pul
Solvent: CDCl3
Temp. 25.0 C / 298.1 K
Operator: barbara
File: 1231-CM-02-117-13C
INOVA-500 "riga"
Relax. delay 4.000 sec
Pulse 52.1 degrees
Acq. time 1.300 sec
Width 29996.3 Hz
1440 repetitions
OBSERVE C13, 125.6674218 MHz
DECOUPLE H1, 499.7732084 MHz
Power 42 dB
on during acquisition
WALTZ-16 modulated
DATA PROCESSING
Line broadening 2.0 Hz
FT size 131072
Total time 96 hr, 39 min, 38 sec
ppm
0
20
40
60
80
100
120
140
160
180
200

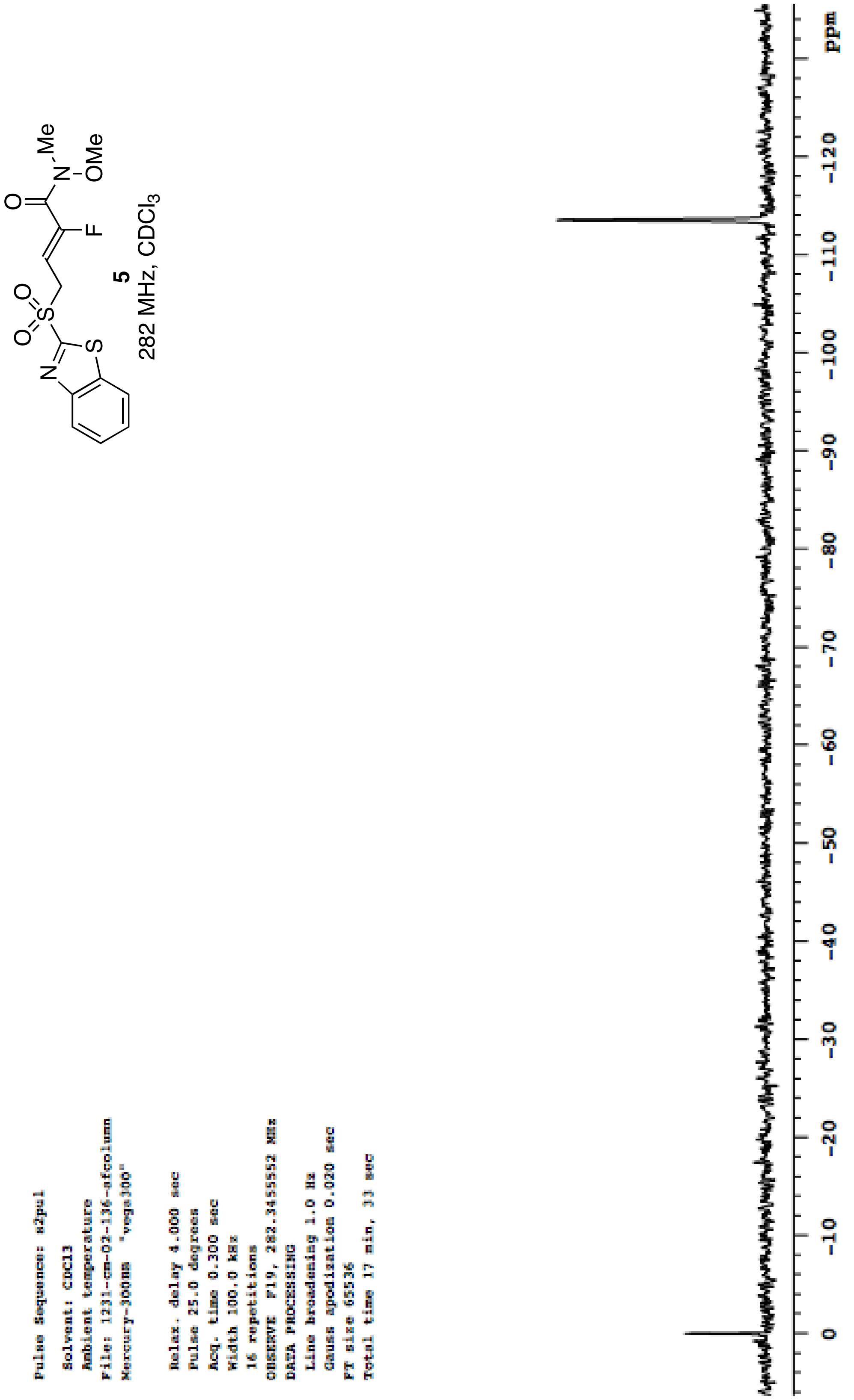
5
282 MHz, CDCl3
Pulse Sequence: s2pul
Solvent: CDCl3
Ambient temperature
File: 1231-cm-02-136-afcolumn
Mercury-300BB "vega300"
Relax. delay 4.000 sec
Pulse 25.0 degrees
Acq. time 0.300 sec
Width 100.0 kHz
16 repetitions
OBSERVE F19, 282.3455552 MHz
DATA PROCESSING
Line broadening 1.0 Hz
Gauss apodization 0.020 sec
FT size 65536
Total time 17 min, 33 sec
0
-10
-20
-30
-40
-50
-60
-70
-80
-90
-100
-110
-120
ppm

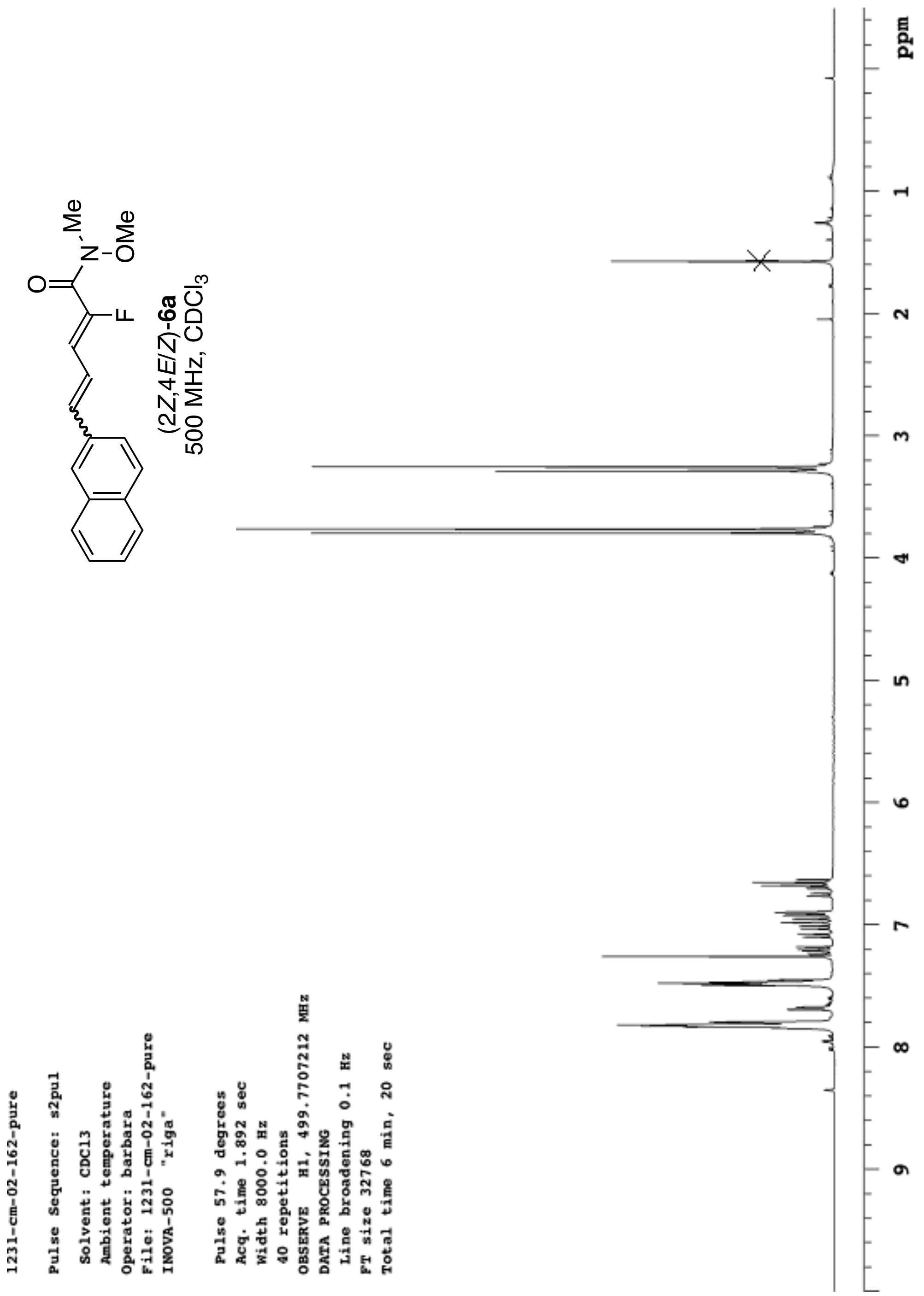
O
N
Me
OMe
F
(2Z,4E/Z)-6a
500 MHz, CDCl3
1231-cm-02-162-pure
Pulse Sequence: s2pul
Solvent: CDCl3
Ambient temperature
Operator: barbara
File: 1231-cm-02-162-pure
INOVA-500 "riga"
Pulse 57.9 degrees
Acq. time 1.892 sec
Width 8000.0 Hz
40 repetitions
OBSERVE H1, 499.7707212 MHz
DATA PROCESSING
Line broadening 0.1 Hz
FT size 32768
Total time 6 min, 20 sec
9
8
7
6
5
4
3
2
1
ppm

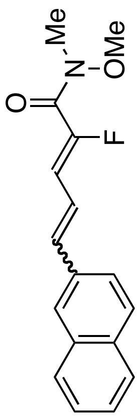

(2*Z*,4*E*/*Z*)-**6a**
282 MHz, $CDCl_3$

Pulse Sequence: s2pul
Solvent: CDCl3
Ambient temperature
File: 1231-CM-02-93-crude
Mercury-300BB "vega300"

Relax. delay 4.000 sec
Pulse 25.0 degrees
Acq. time 0.300 sec
Width 100.0 kHz
72 repetitions
OBSERVE F19, 282.3455583 MHz
DATA PROCESSING
Line broadening 4.0 Hz
FT size 65536
Total time 17 min, 33 sec

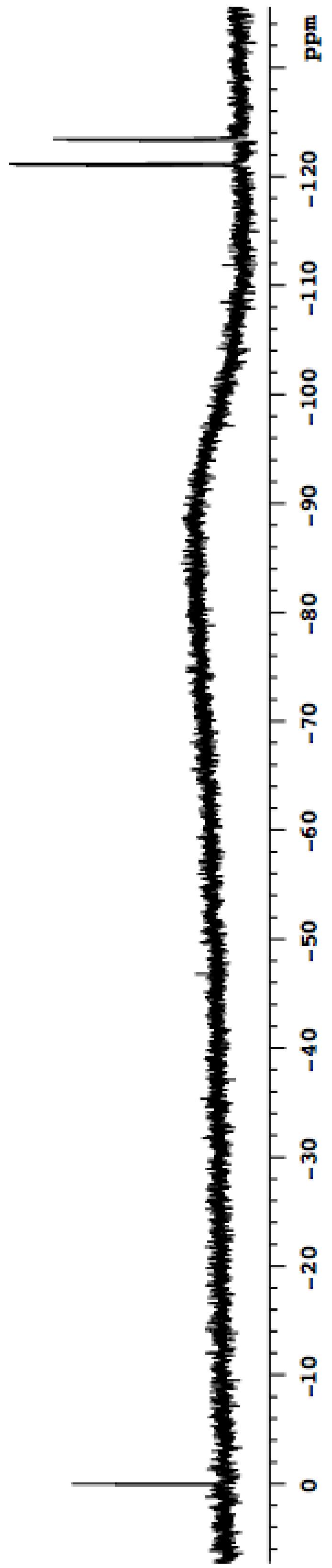

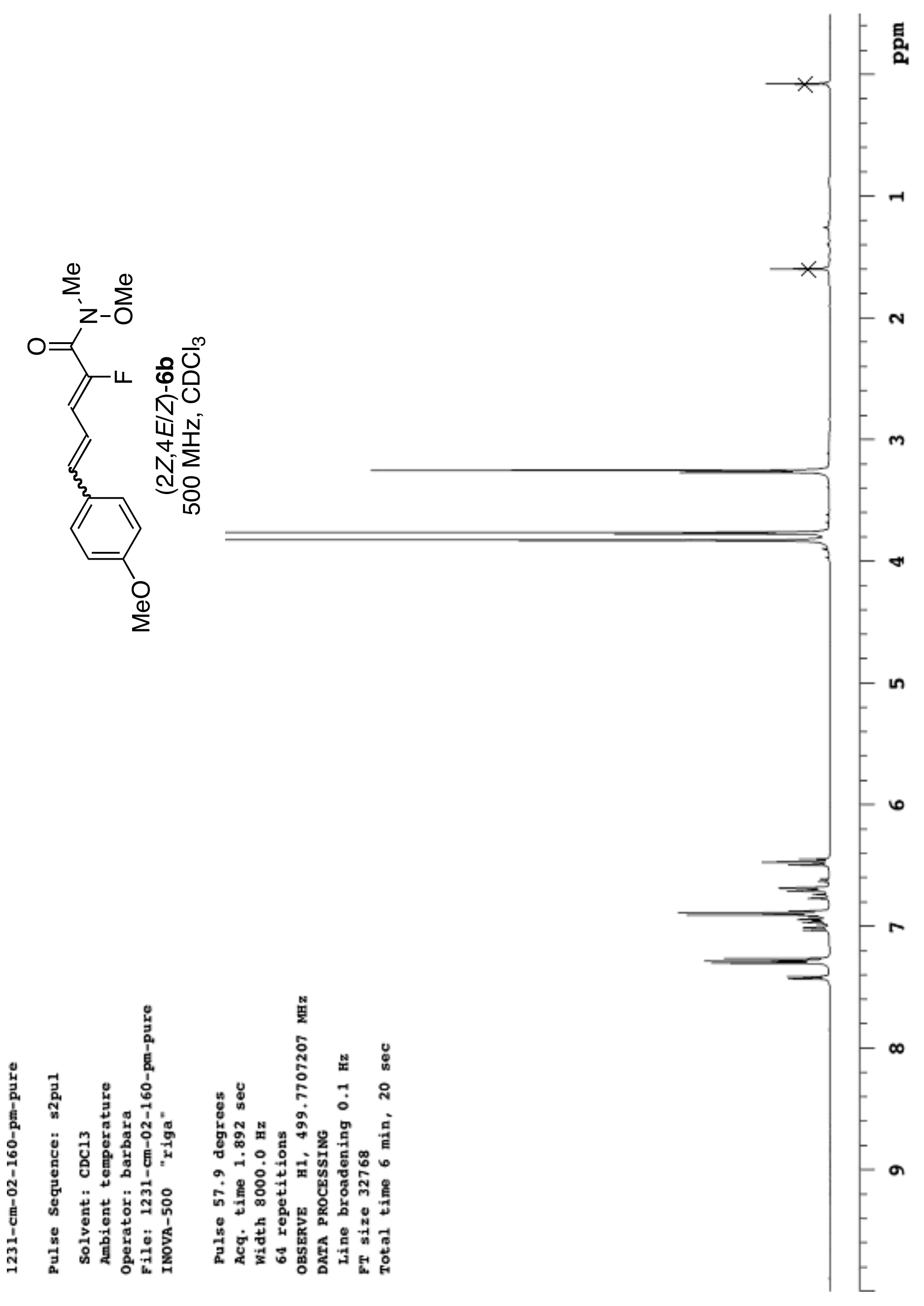
MeO
O
N
Me
OMe
F
(2Z,4E/Z)-6b
500 MHz, CDCl3
1231-cm-02-160-pm-pure
Pulse Sequence: s2pul
Solvent: CDCl3
Ambient temperature
Operator: barbara
File: 1231-cm-02-160-pm-pure
INOVA-500 "riga"
Pulse 57.9 degrees
Acq. time 1.892 sec
Width 8000.0 Hz
64 repetitions
OBSERVE H1, 499.7707207 MHz
DATA PROCESSING
Line broadening 0.1 Hz
FT size 32768
Total time 6 min, 20 sec
9
8
7
6
5
4
3
2
1
ppm

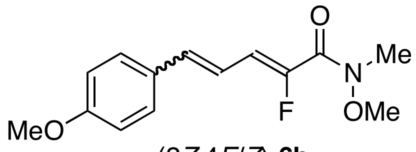

(2*Z*,4*E*/*Z*)-**6b**
282 MHz, $CDCl_3$

Pulse Sequence: s2pul
Solvent: CDCl3
Ambient temperature
File: 1231-cm-03-160-pure-19F
Mercury-300BB "vega300"

Relax. delay 4.000 sec
Pulse 25.0 degrees
Acq. time 0.300 sec
Width 100.0 kHz
64 repetitions
OBSERVE F19, 282.3455583 MHz
DATA PROCESSING
Line broadening 1.0 Hz
FT size 65536
Total time 17 min, 33 sec

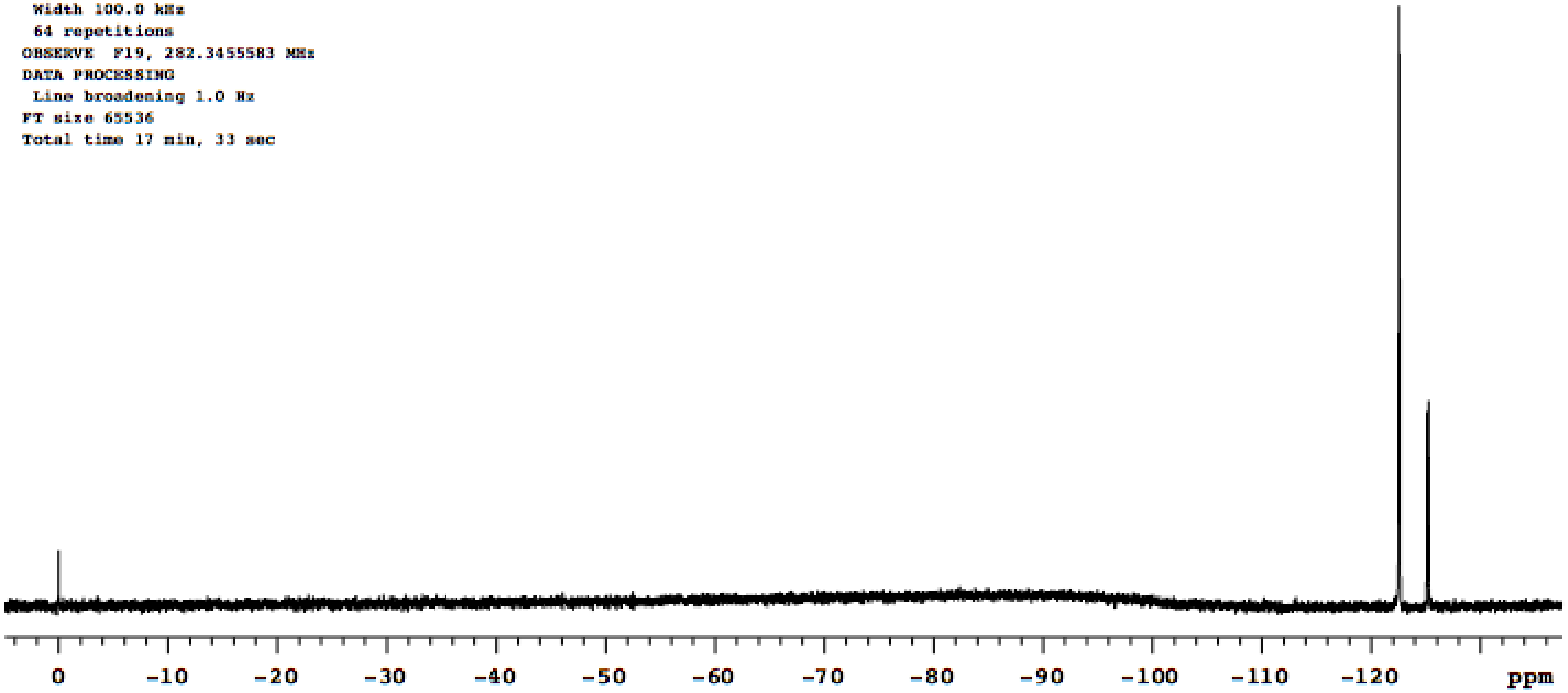

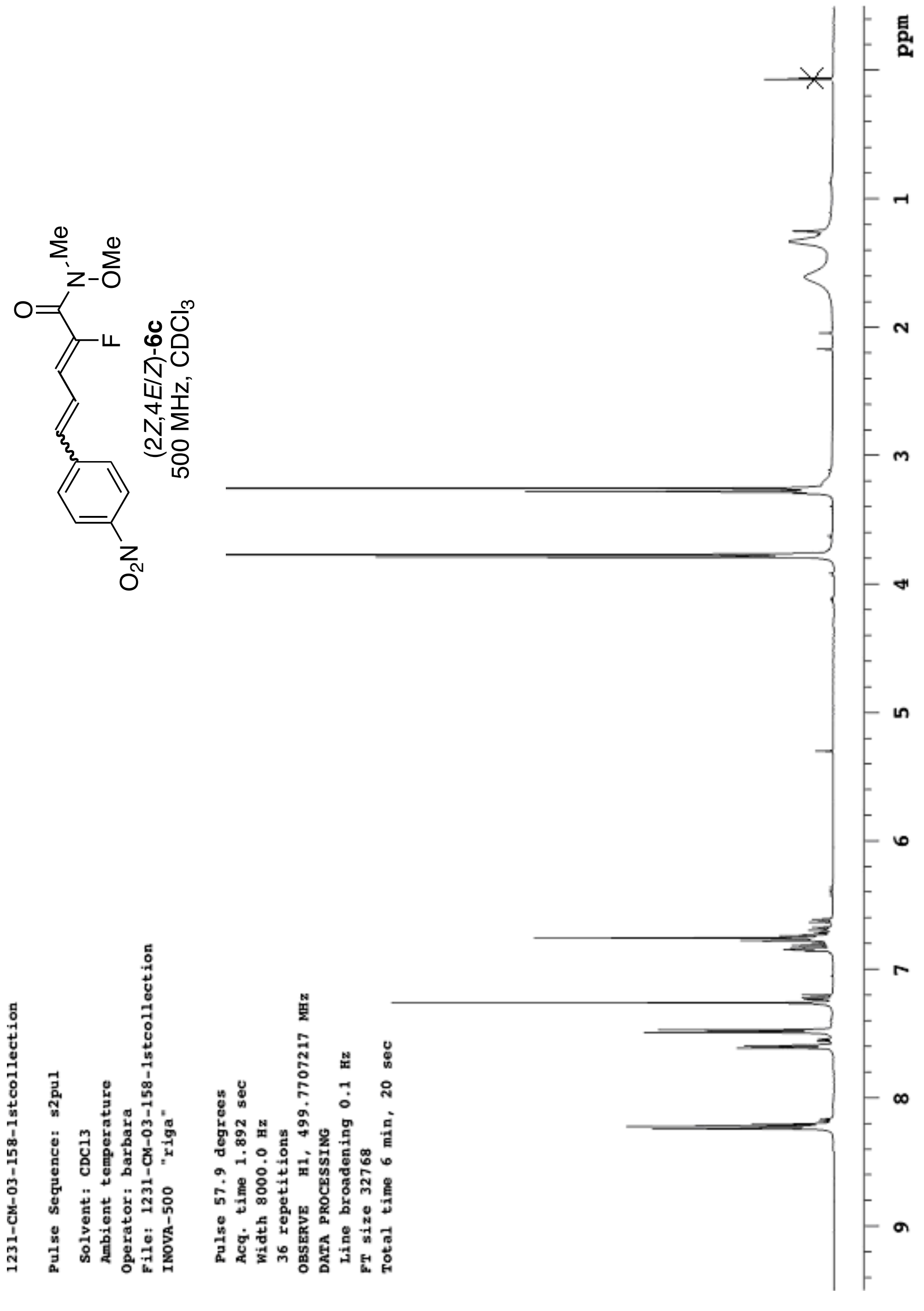
1231-CM-03-158-1stcollection
Pulse Sequence: s2pul
Solvent: CDCl3
Ambient temperature
Operator: barbara
File: 1231-CM-03-158-1stcollection
INOVA-500 "riga"
Pulse 57.9 degrees
Acq. time 1.892 sec
Width 8000.0 Hz
36 repetitions
OBSERVE H1, 499.7707217 MHz
DATA PROCESSING
Line broadening 0.1 Hz
FT size 32768
Total time 6 min, 20 sec
(2Z,4E/Z)-6c
500 MHz, CDCl3
9
8
7
6
5
4
3
2
1
ppm

Pulse Sequence: s2pul

Solvent: CDCl3
Ambient temperature
File: 1231-cm-03-158-1stcollection-afPPTLC
Mercury-300BB "vega300"

Relax. delay 4.000 sec
Pulse 25.0 degrees
Acq. time 0.300 sec
Width 100.0 kHz
44 repetitions
OBSERVE F19, 282.3455583 MHz
DATA PROCESSING
Line broadening 1.0 Hz
FT size 65536
Total time 17 min, 33 sec

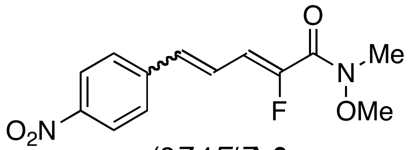

(2*Z*,4*E*/*Z*)-**6c**
282 MHz, $CDCl_3$

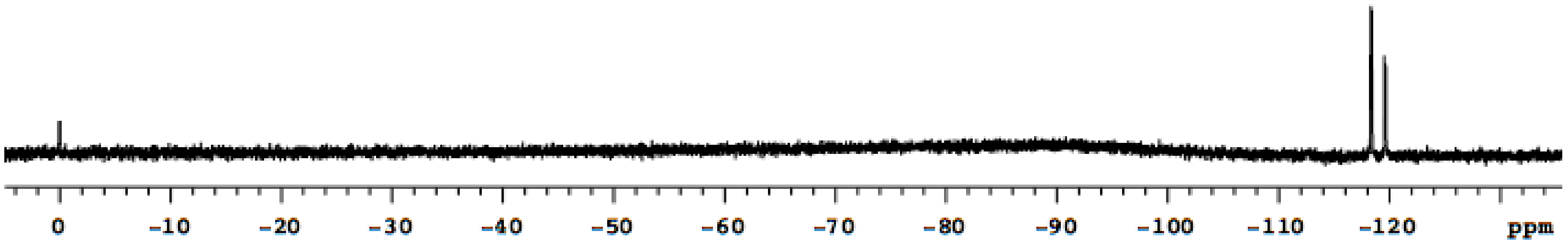

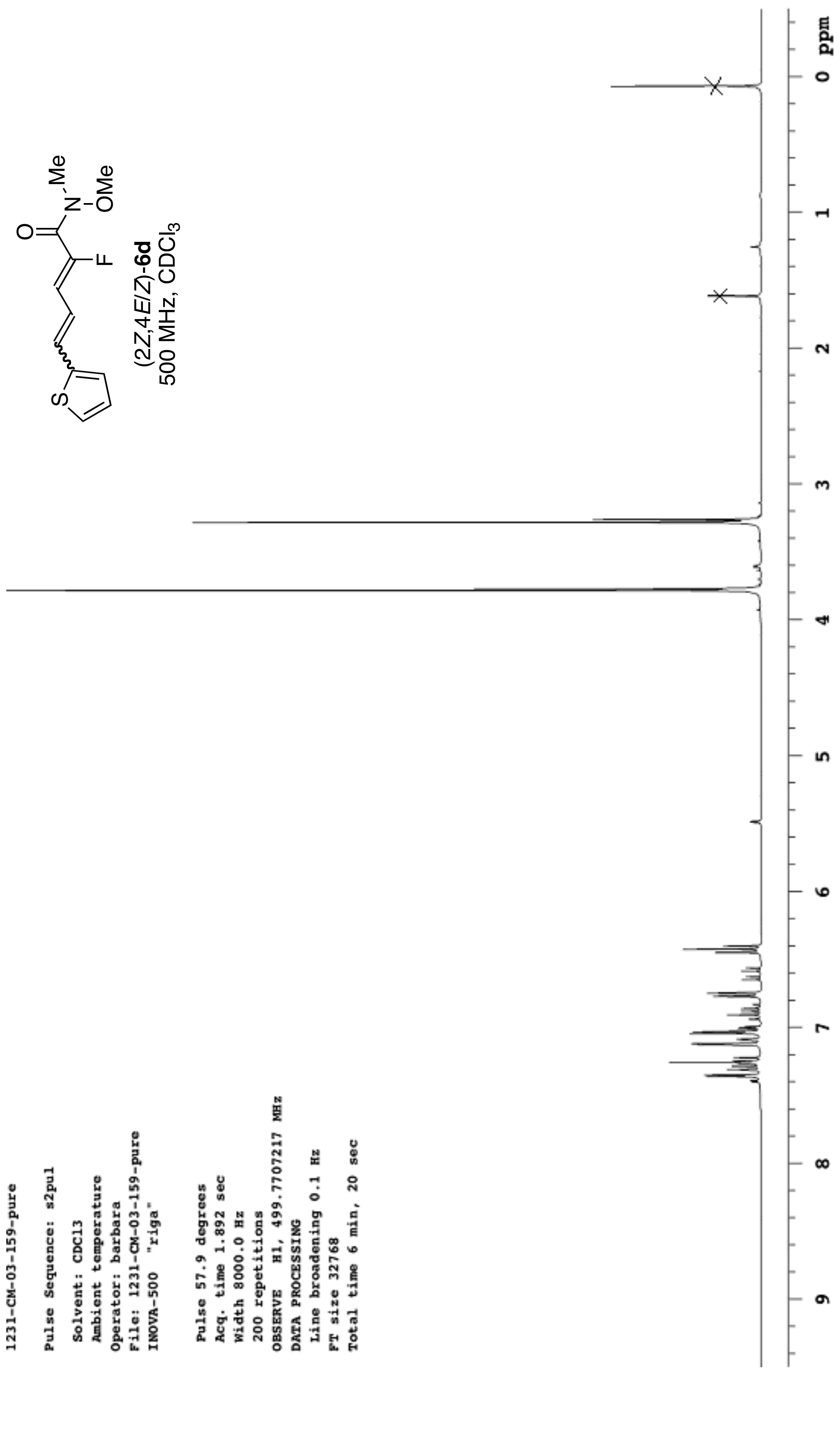
1231-CM-03-159-pure
Pulse Sequence: s2pul
Solvent: CDCl3
Ambient temperature
Operator: barbara
File: 1231-CM-03-159-pure
INOVA-500 "riga"
Pulse 57.9 degrees
Acq. time 1.892 sec
Width 8000.0 Hz
200 repetitions
OBSERVE H1, 499.7707217 MHz
DATA PROCESSING
Line broadening 0.1 Hz
FT size 32768
Total time 6 min, 20 sec
O
N
Me
OMe
F
S
(2Z,4E/Z)-6d
500 MHz, CDCl3
9
8
7
6
5
4
3
2
1
0 ppm

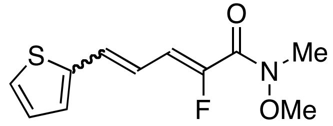

Crude mixture of
(2*Z*,4*E*/*Z*)-**6d**
282 MHz, $CDCl_3$

Pulse Sequence: s2pul

Solvent: CDCl3
Ambient temperature
File: 1231-cm-03-159-rm
Mercury-300BB "vega300"

Relax. delay 4.000 sec
Pulse 25.0 degrees
Acq. time 0.300 sec
Width 100.0 kHz
188 repetitions
OBSERVE F19, 282.3454332 MHz
DATA PROCESSING
Line broadening 1.0 Hz
FT size 65536
Total time 17 min, 33 sec

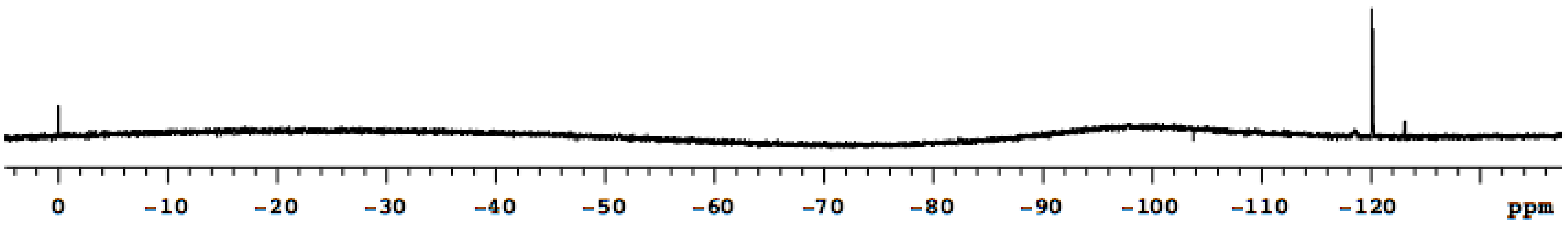

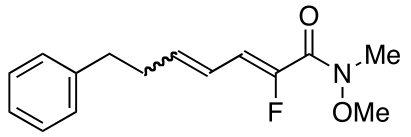

(2*Z*,4*E*/*Z*)-**6e**
500 MHz, $CDCl_3$

1231-cm-03-161-pure

Pulse Sequence: s2pul

Solvent: CDCl3
Ambient temperature
Operator: barbara
File: 1231-cm-03-161-pure
INOVA-500 "riga"

Pulse 57.9 degrees
Acq. time 1.892 sec
Width 8000.0 Hz
200 repetitions
OBSERVE H1, 499.7707212 MHz
DATA PROCESSING
Line broadening 0.1 Hz
FT size 32768
Total time 6 min, 20 sec

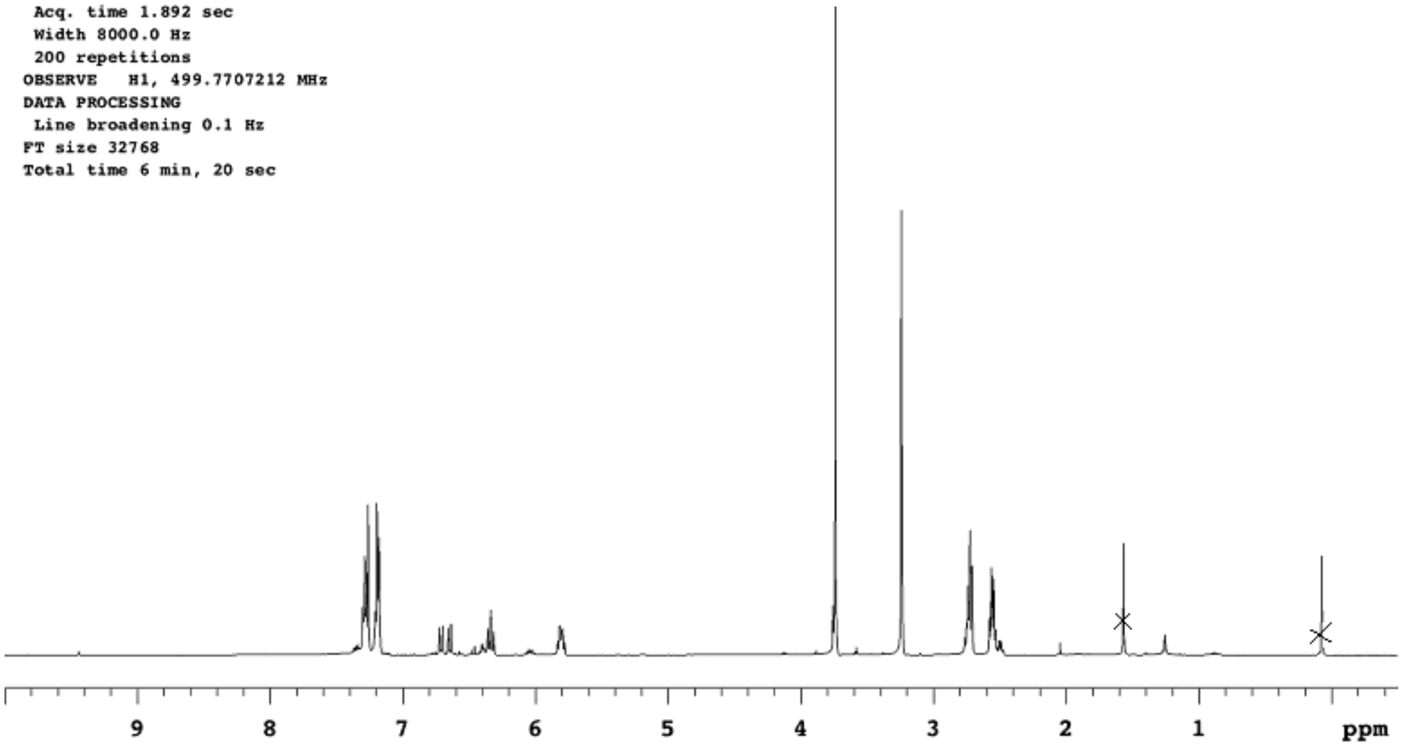

Pulse Sequence: s2pul

Solvent: CDCl3
Ambient temperature
File: 1231-cm-03-161-rm
Mercury-300BB "vega300"

Relax. delay 4.000 sec
Pulse 25.0 degrees
Acq. time 0.300 sec
Width 100.0 kHz
24 repetitions
OBSERVE F19, 282.3455552 MHz
DATA PROCESSING
Line broadening 1.0 Hz
FT size 65536
Total time 17 min, 33 sec

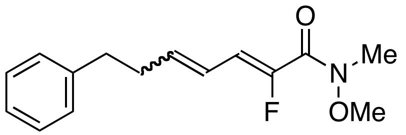

Crude mixture of
(2*Z*,4*E*/*Z*)-**6e**
282 MHz, $CDCl_3$

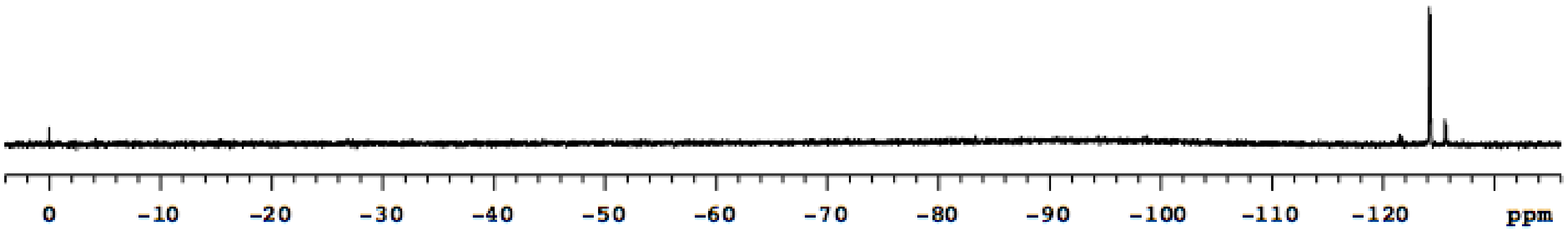

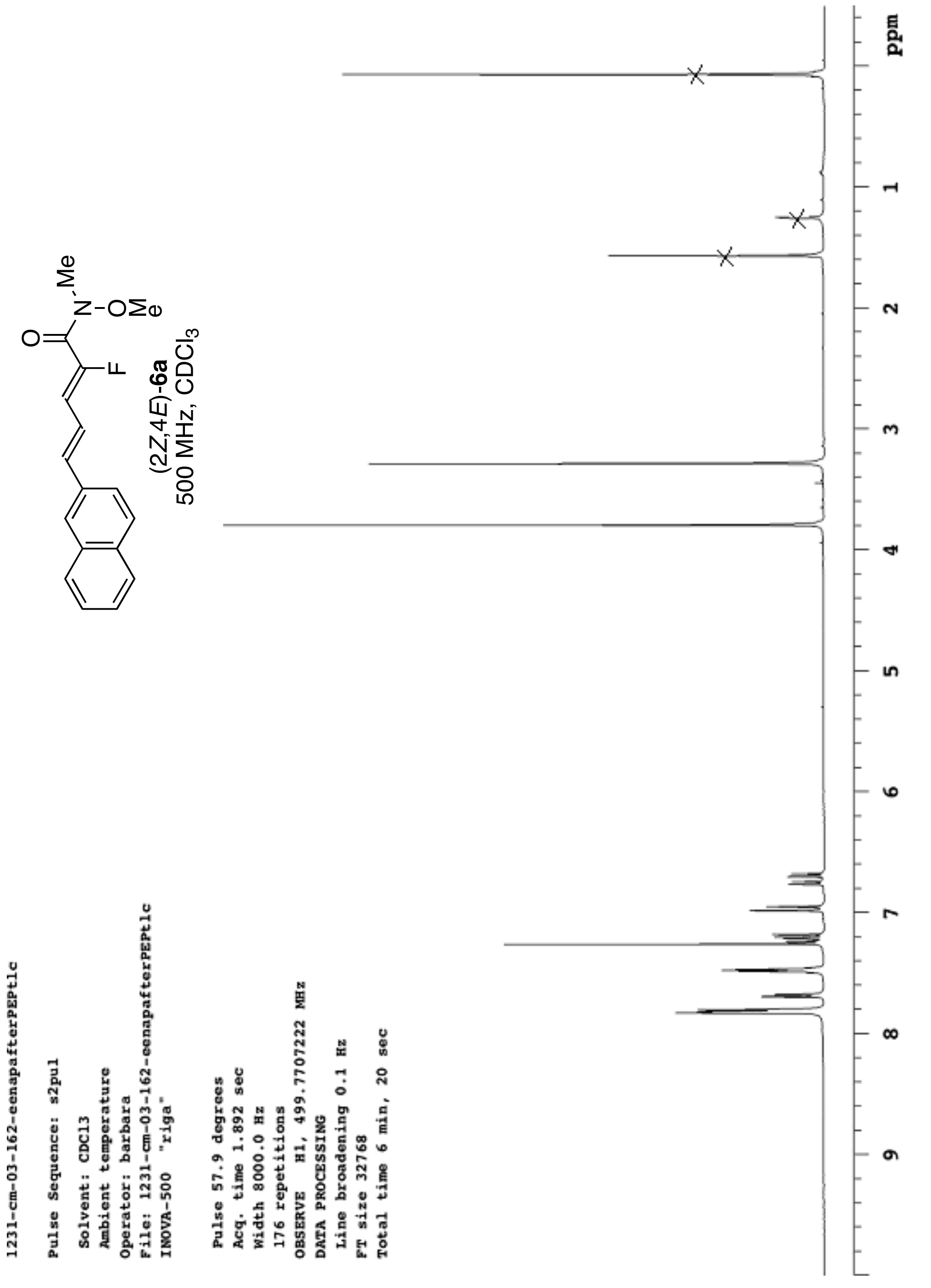
O
N
Me
O
Me
F
(2Z,4E)-6a
500 MHz, CDCl3
1231-cm-03-162-eenapafterPEPtlc
Pulse Sequence: s2pul
Solvent: CDCl3
Ambient temperature
Operator: barbara
File: 1231-cm-03-162-eenapafterPEPtlc
INOVA-500 "riga"
Pulse 57.9 degrees
Acq. time 1.892 sec
Width 8000.0 Hz
176 repetitions
OBSERVE H1, 499.7707222 MHz
DATA PROCESSING
Line broadening 0.1 Hz
FT size 32768
Total time 6 min, 20 sec
9
8
7
6
5
4
3
2
1
ppm

```
1231-CM-03-162-EEnaph-13C

Pulse Sequence: s2pul

 Solvent: CDCl3
 Temp. 25.0 C / 298.1 K
Operator: barbara
File: 1231-CM-03-162-EEnaphtha-13C
INOVA-500  "riga"

 Relax. delay 4.000 sec
 Pulse 52.1 degrees
 Acq. time 1.300 sec
 Width 29996.3 Hz
 10548 repetitions
OBSERVE  C13, 125.6674182 MHz
DECOUPLE  H1, 499.7732084 MHz
 Power 42 dB
 on during acquisition
 WALTZ-16 modulated
DATA PROCESSING
 Line broadening 2.0 Hz
FT size 131072
Total time 96 hr, 39 min, 38 sec
```

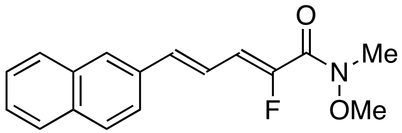

(2*Z*,4*E*)-**6a**
125 MHz, $CDCl_3$

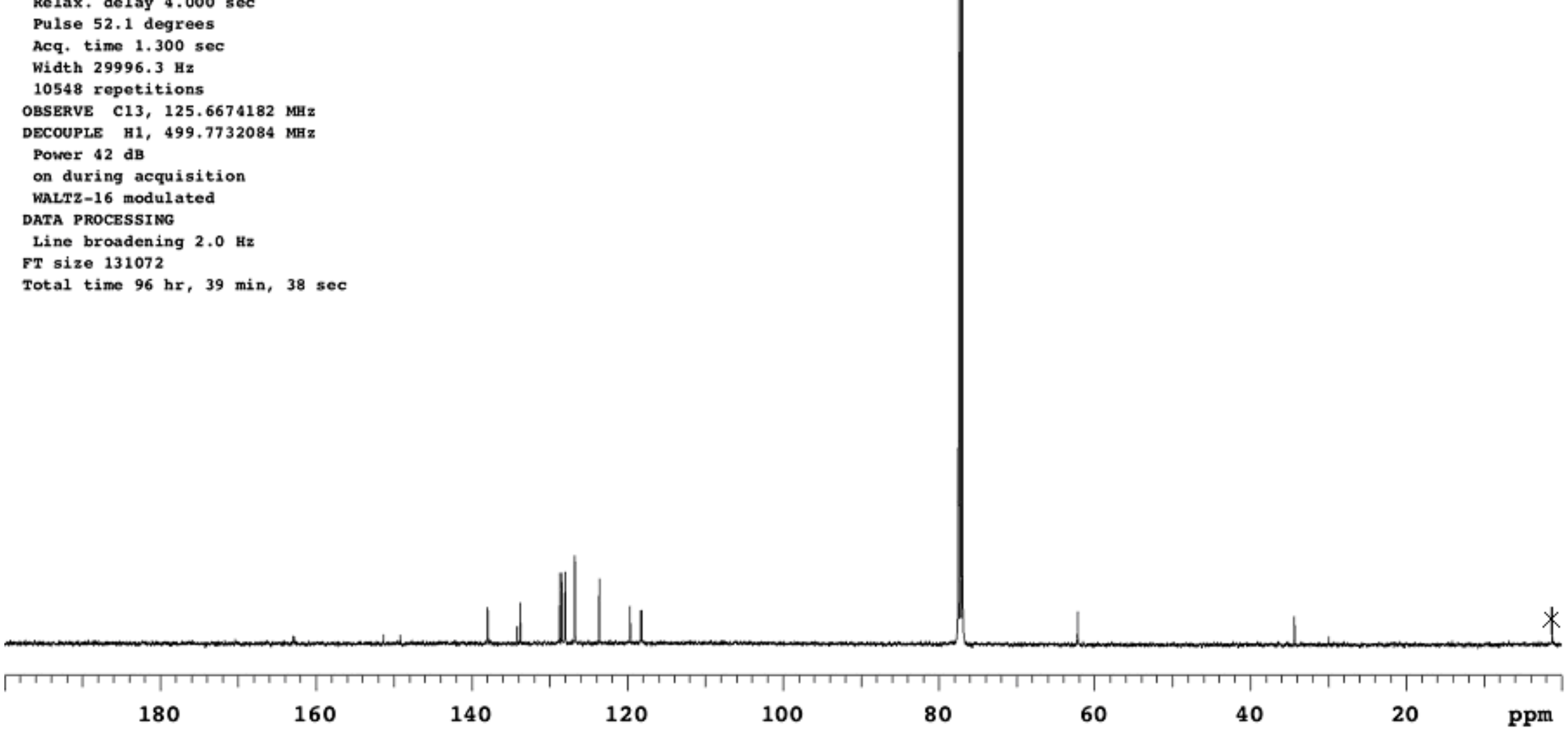

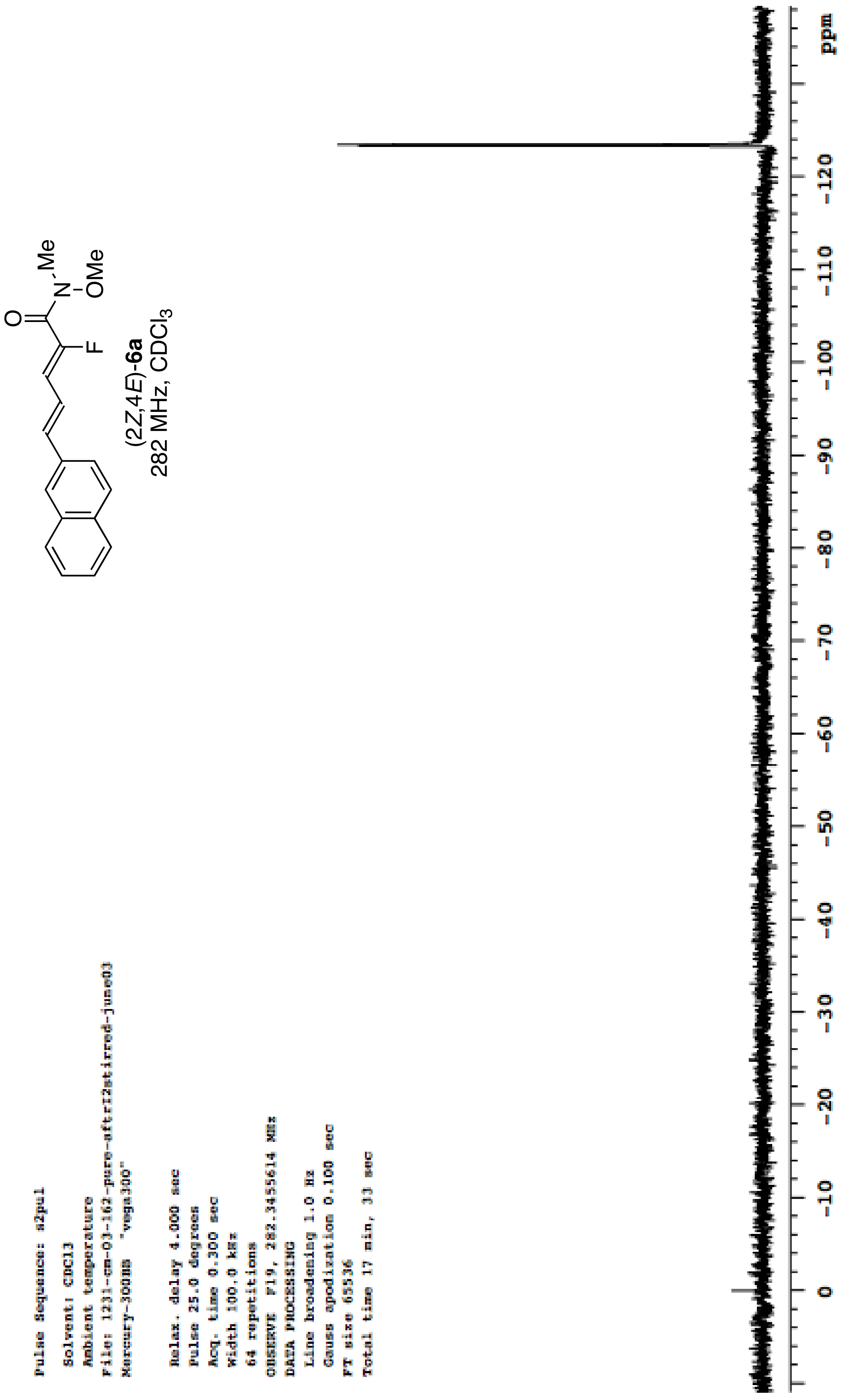

(2*Z*,4*E*)-**6a**
282 MHz, $CDCl_3$

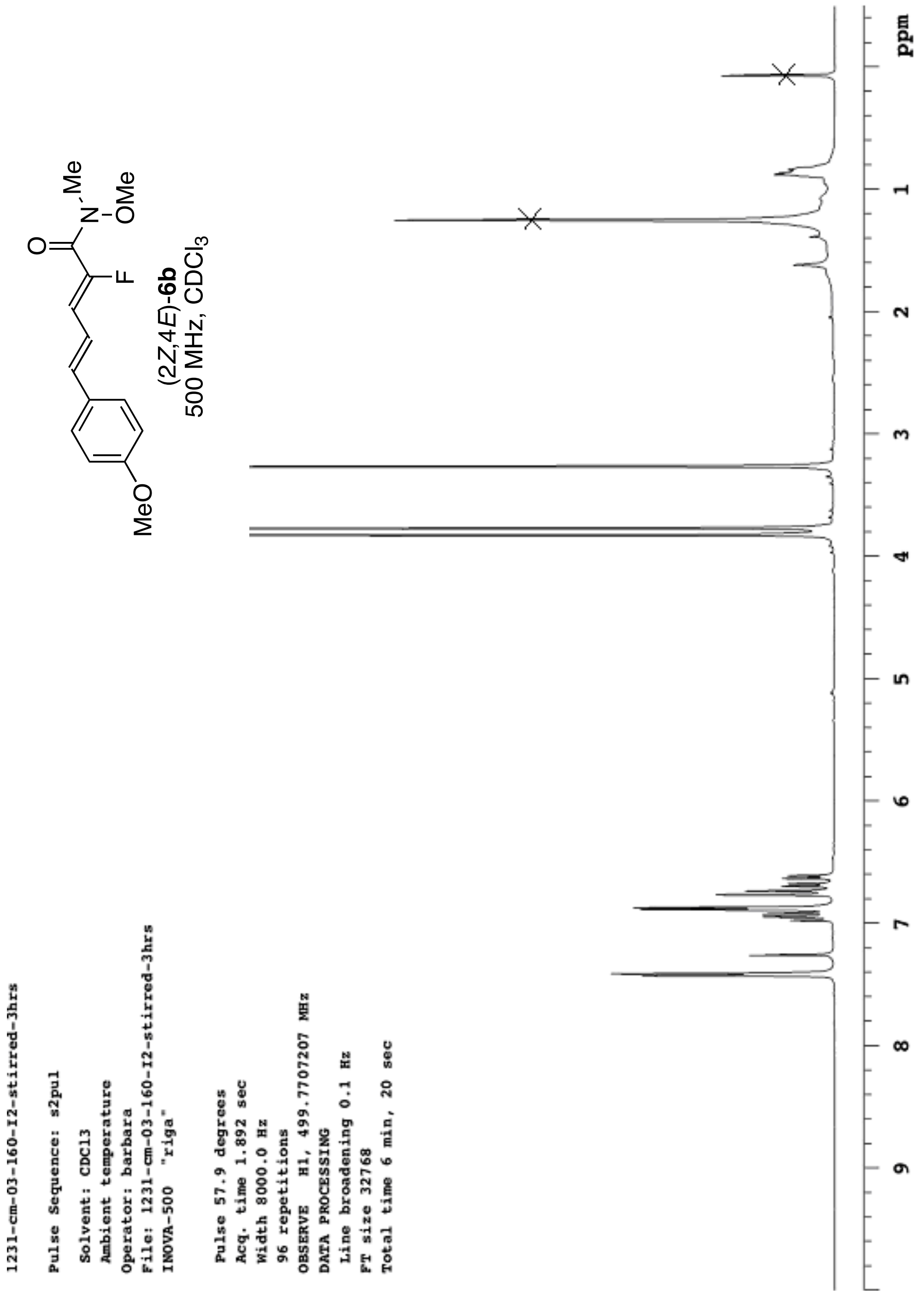
O
N
Me
OMe
F
MeO
(2Z,4E)-6b
500 MHz, CDCl3
1231-cm-03-160-I2-stirred-3hrs
Pulse Sequence: s2pul
Solvent: CDCl3
Ambient temperature
Operator: barbara
File: 1231-cm-03-160-I2-stirred-3hrs
INOVA-500 "riga"
Pulse 57.9 degrees
Acq. time 1.892 sec
Width 8000.0 Hz
96 repetitions
OBSERVE H1, 499.7707207 MHz
DATA PROCESSING
Line broadening 0.1 Hz
FT size 32768
Total time 6 min, 20 sec
ppm
1
2
3
4
5
6
7
8
9

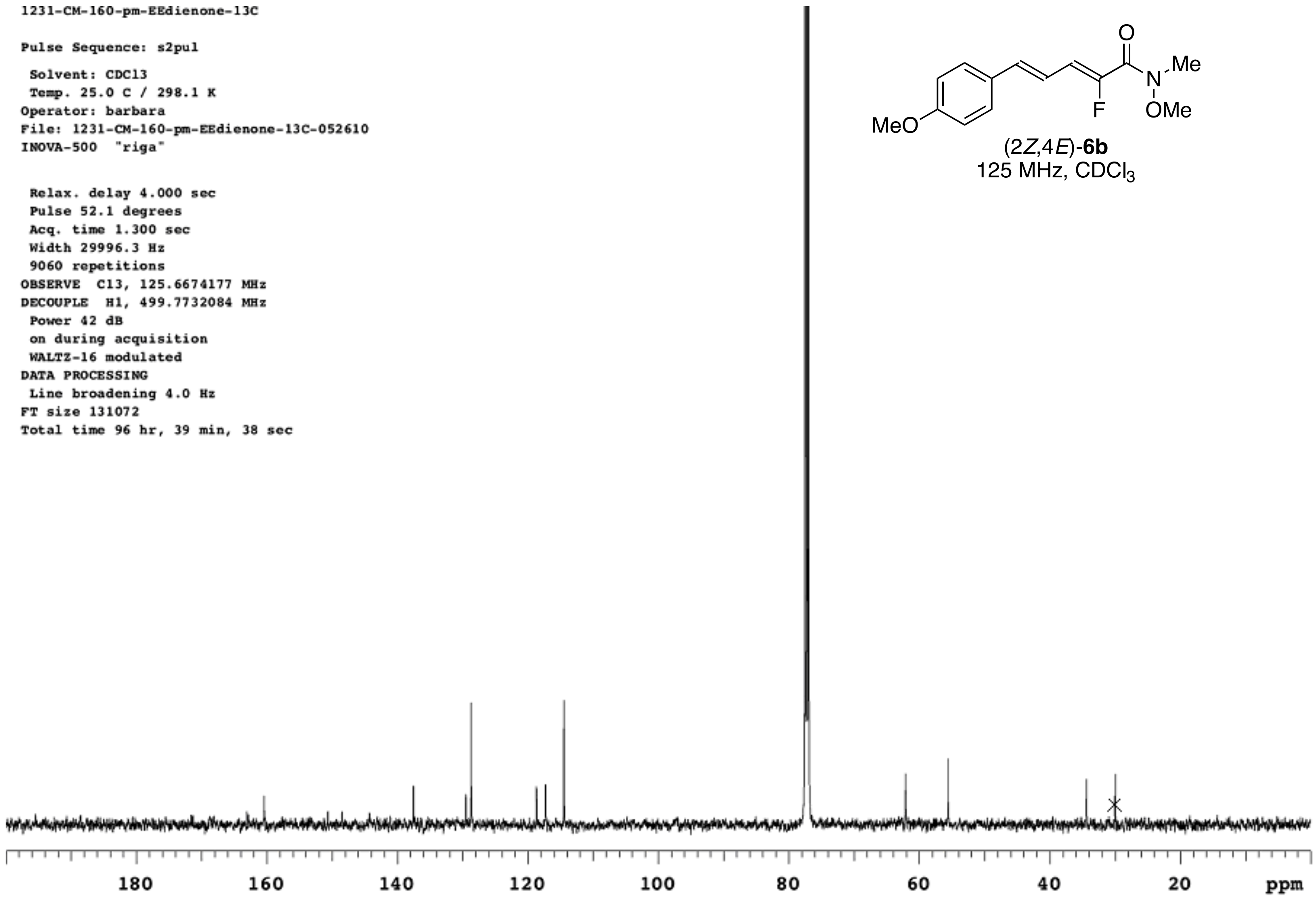
1231-CM-160-pm-EEdienone-13C
Pulse Sequence: s2pul
Solvent: CDCl3
Temp. 25.0 C / 298.1 K
Operator: barbara
File: 1231-CM-160-pm-EEdienone-13C-052610
INOVA-500 "riga"
Relax. delay 4.000 sec
Pulse 52.1 degrees
Acq. time 1.300 sec
Width 29996.3 Hz
9060 repetitions
OBSERVE C13, 125.6674177 MHz
DECOUPLE H1, 499.7732084 MHz
Power 42 dB
on during acquisition
WALTZ-16 modulated
DATA PROCESSING
Line broadening 4.0 Hz
FT size 131072
Total time 96 hr, 39 min, 38 sec
MeO
O
N
Me
F
OMe
(2Z,4E)-6b
125 MHz, CDCl3
180
160
140
120
100
80
60
40
20
ppm

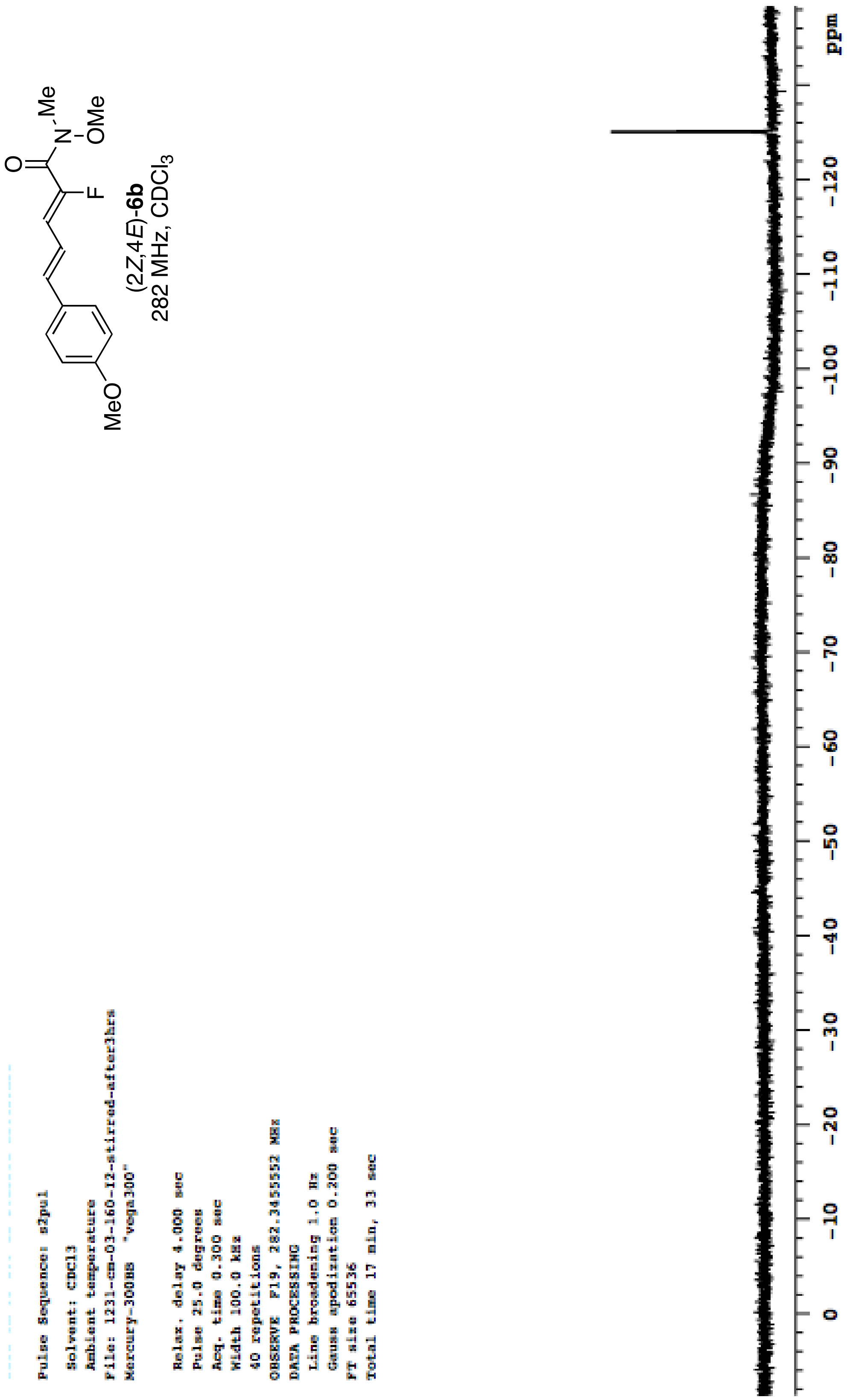
MeO
O
N
Me
OMe
F
(2Z,4E)-6b
282 MHz, CDCl3
Pulse Sequence: s2pul
Solvent: CDCl3
Ambient temperature
File: 1231-cm-03-160-I2-stirred-after3hrs
Mercury-300BB "vega300"
Relax. delay 4.000 sec
Pulse 25.0 degrees
Acq. time 0.300 sec
Width 100.0 kHz
40 repetitions
OBSERVE F19, 282.3455552 MHz
DATA PROCESSING
Line broadening 1.0 Hz
Gauss apodization 0.200 sec
FT size 65536
Total time 17 min, 33 sec
-120
-110
-100
-90
-80
-70
-60
-50
-40
-30
-20
-10
0
ppm

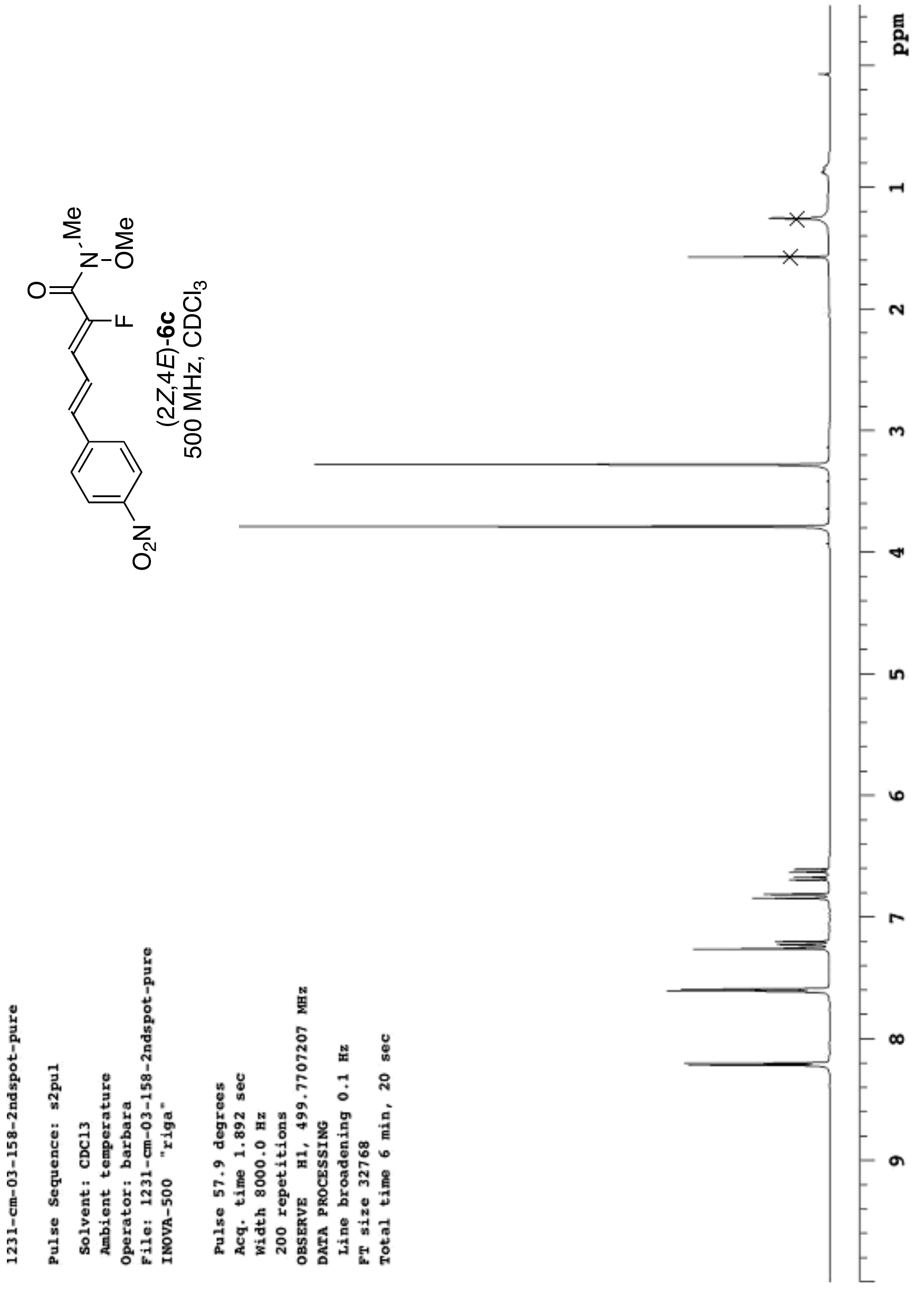

1231-cm-03-158-2ndspot-pure
Pulse Sequence: s2pul
Solvent: CDCl3
Ambient temperature
Operator: barbara
File: 1231-cm-03-158-2ndspot-pure
INOVA-500 "riga"
Pulse 57.9 degrees
Acq. time 1.892 sec
Width 8000.0 Hz
200 repetitions
OBSERVE H1, 499.7707207 MHz
DATA PROCESSING
Line broadening 0.1 Hz
FT size 32768
Total time 6 min, 20 sec
O
N
Me
OMe
F
O2N
(2Z,4E)-6c
500 MHz, CDCl3
ppm
1
2
3
4
5
6
7
8
9

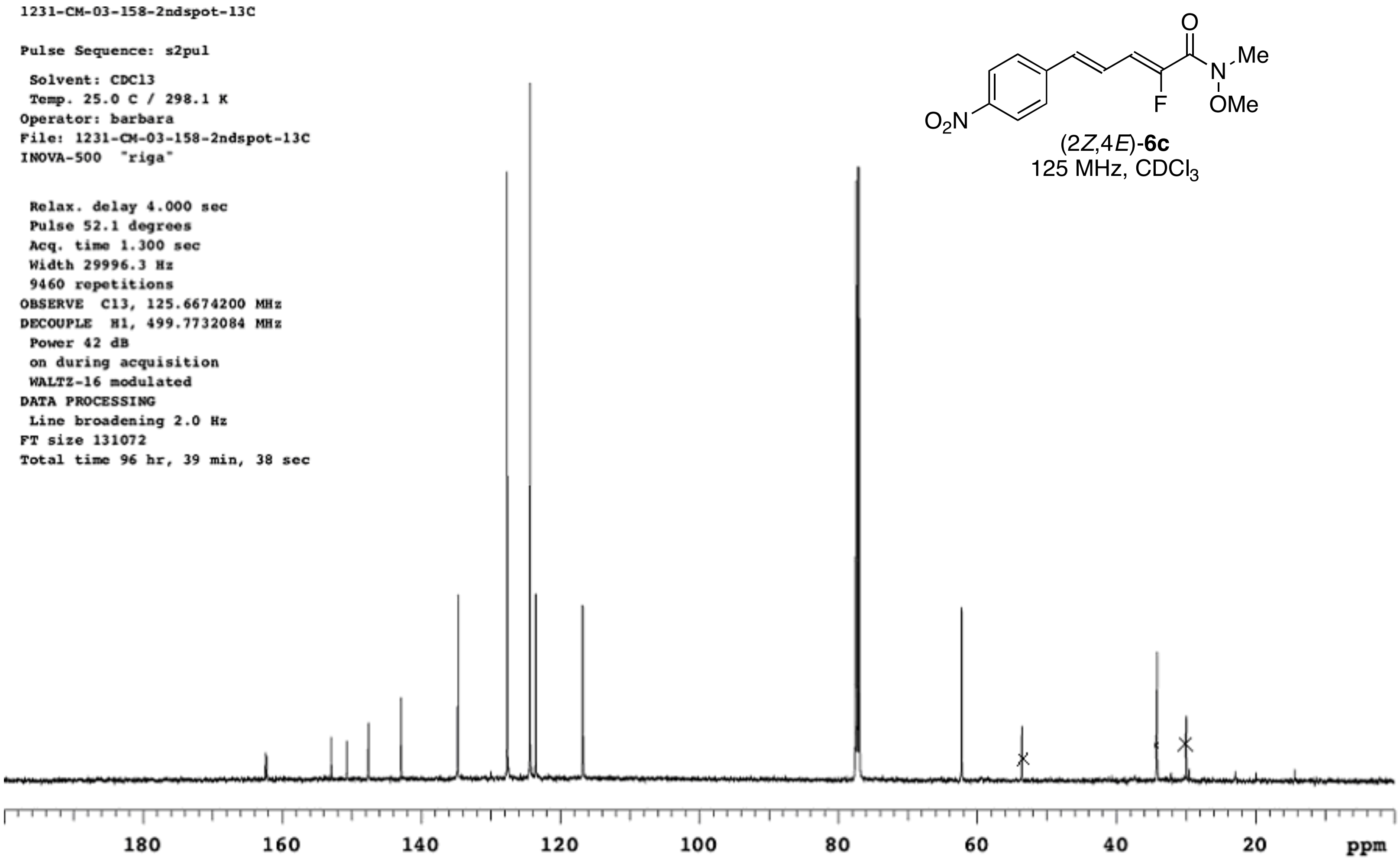
1231-CM-03-158-2ndspot-13C
Pulse Sequence: s2pul
Solvent: CDCl3
Temp. 25.0 C / 298.1 K
Operator: barbara
File: 1231-CM-03-158-2ndspot-13C
INOVA-500 "riga"
Relax. delay 4.000 sec
Pulse 52.1 degrees
Acq. time 1.300 sec
Width 29996.3 Hz
9460 repetitions
OBSERVE C13, 125.6674200 MHz
DECOUPLE H1, 499.7732084 MHz
Power 42 dB
on during acquisition
WALTZ-16 modulated
DATA PROCESSING
Line broadening 2.0 Hz
FT size 131072
Total time 96 hr, 39 min, 38 sec
O
Me
N
O2N
F
OMe
(2Z,4E)-6c
125 MHz, CDCl3
180
160
140
120
100
80
60
40
20
ppm

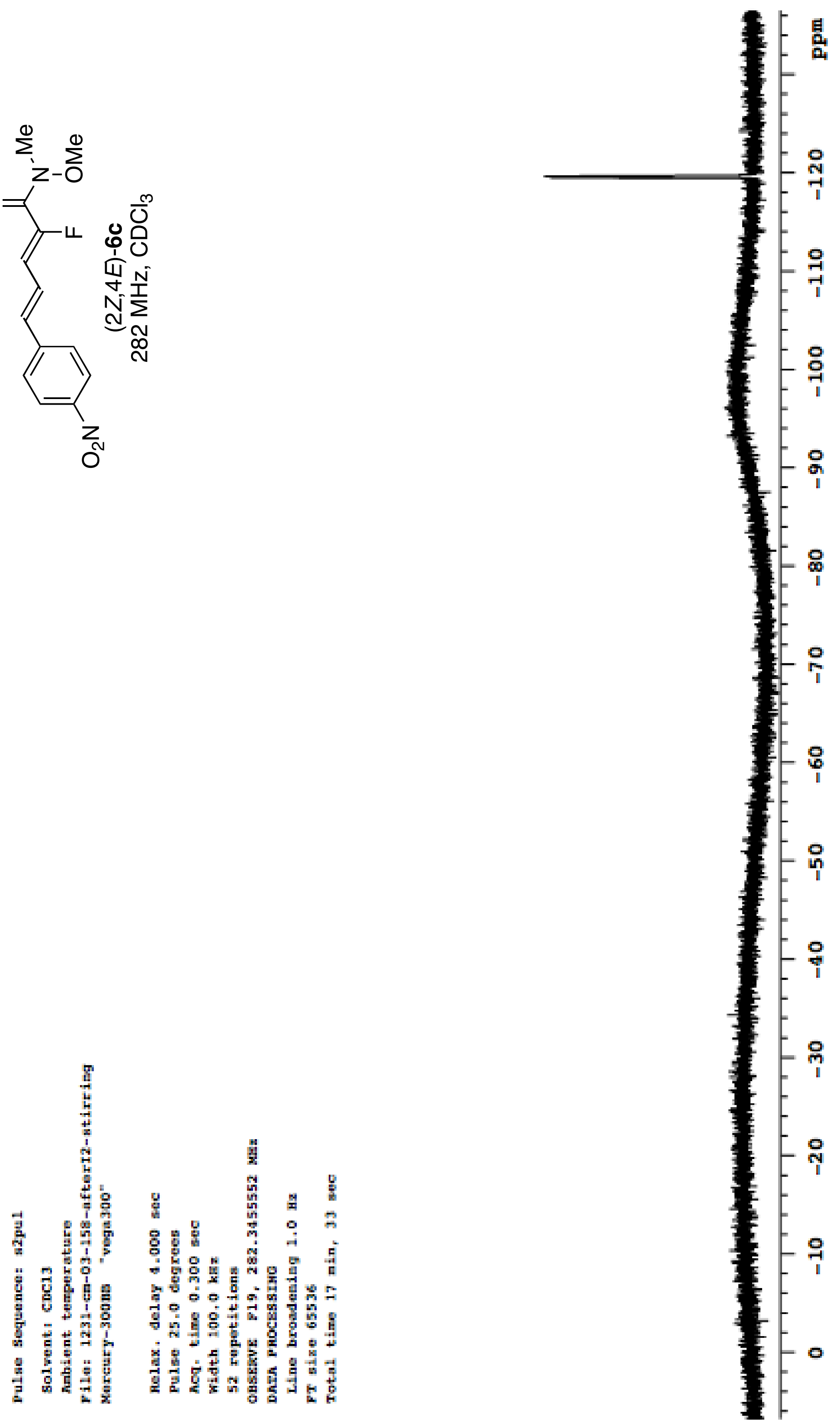

(2Z,4E)-6c
282 MHz, CDCl3
O2N
O
N
Me
OMe
F
Pulse Sequence: s2pul
Solvent: CDCl3
Ambient temperature
File: 1231-cm-03-158-afterI2-stirring
Mercury-300BB "vega300"
Relax. delay 4.000 sec
Pulse 25.0 degrees
Acq. time 0.300 sec
Width 100.0 kHz
52 repetitions
OBSERVE F19, 282.3455552 MHz
DATA PROCESSING
Line broadening 1.0 Hz
FT size 65536
Total time 17 min, 33 sec
ppm
-120
-110
-100
-90
-80
-70
-60
-50
-40
-30
-20
-10
0

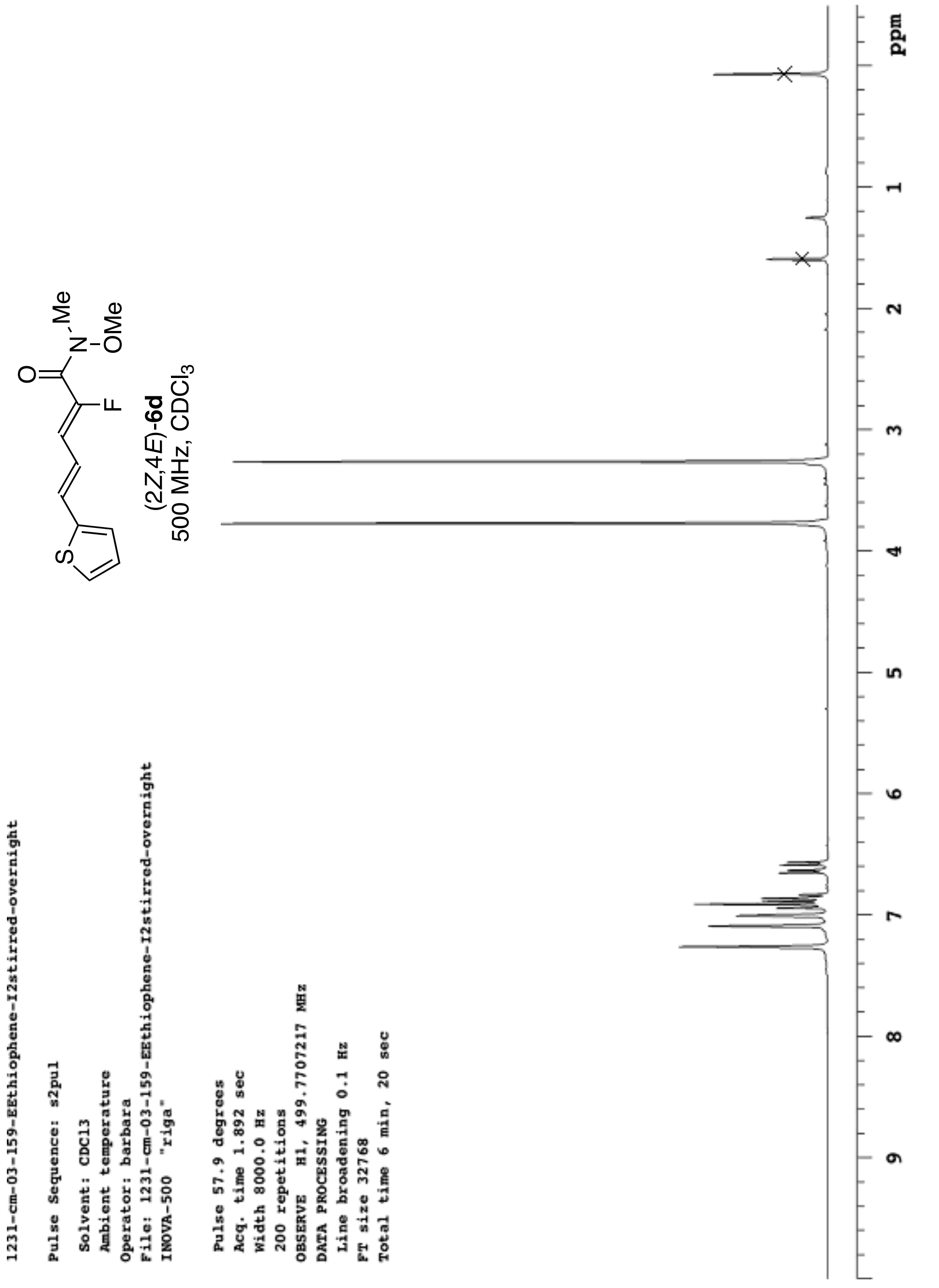
1231-cm-03-159-EEthiophene-I2stirred-overnight
Pulse Sequence: s2pul
Solvent: CDCl3
Ambient temperature
Operator: barbara
File: 1231-cm-03-159-EEthiophene-I2stirred-overnight
INOVA-500 "riga"
Pulse 57.9 degrees
Acq. time 1.892 sec
Width 8000.0 Hz
200 repetitions
OBSERVE H1, 499.7707217 MHz
DATA PROCESSING
Line broadening 0.1 Hz
FT size 32768
Total time 6 min, 20 sec
O
N
Me
OMe
F
S
(2Z,4E)-6d
500 MHz, CDCl3
ppm
1
2
3
4
5
6
7
8
9

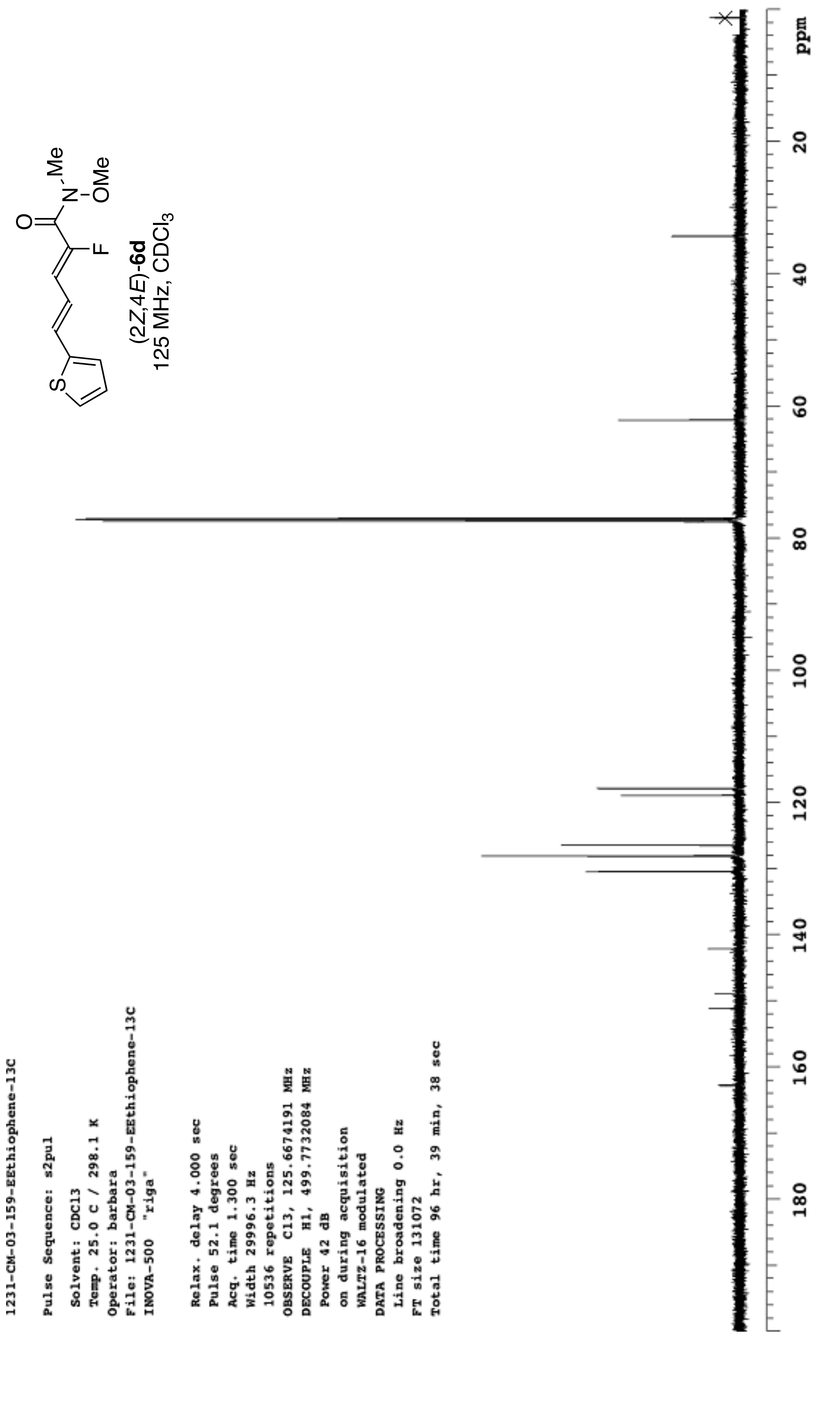
(2Z,4E)-6d
125 MHz, CDCl3
1231-CM-03-159-EEthiophene-13C
Pulse Sequence: s2pul
Solvent: CDCl3
Temp. 25.0 C / 298.1 K
Operator: barbara
File: 1231-CM-03-159-EEthiophene-13C
INOVA-500 "riga"
Relax. delay 4.000 sec
Pulse 52.1 degrees
Acq. time 1.300 sec
Width 29996.3 Hz
10536 repetitions
OBSERVE C13, 125.6674191 MHz
DECOUPLE H1, 499.7732084 MHz
Power 42 dB
on during acquisition
WALTZ-16 modulated
DATA PROCESSING
Line broadening 0.0 Hz
FT size 131072
Total time 96 hr, 39 min, 38 sec
ppm
20
40
60
80
100
120
140
160
180

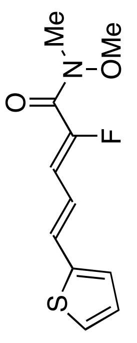

(2*Z*,4*E*)-**6d**
282 MHz, $CDCl_3$

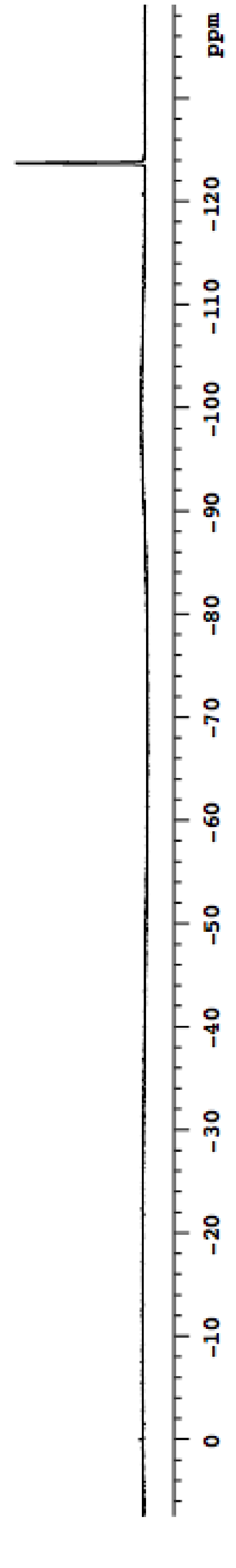

Supplement: Supplementary file 1 [file molecules-19-04418-s001.pdf]
